# Activation of Glycyl Radical Enzymes—Multiscale Modeling Insights into Catalysis and Radical Control in a Pyruvate Formate-Lyase-Activating Enzyme

**DOI:** 10.1021/acs.jcim.2c00362

**Published:** 2022-06-30

**Authors:** Marko Hanževački, Anna K. Croft, Christof M. Jäger

**Affiliations:** Department of Chemical and Environmental Engineering, University of Nottingham, Nottingham NG7 2RD, U.K.

## Abstract

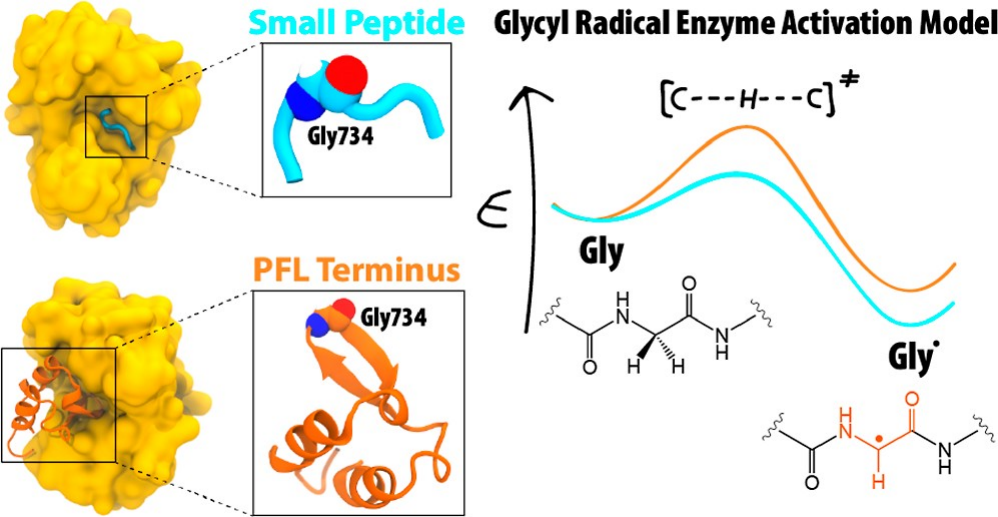

Pyruvate formate-lyase (PFL) is a glycyl radical enzyme (GRE) playing a pivotal role in the metabolism of strict and facultative anaerobes. Its activation is carried out by a PFL-activating enzyme, a member of the radical S-adenosylmethionine (rSAM) superfamily of metalloenzymes, which introduces a glycyl radical into the Gly radical domain of PFL. The activation mechanism is still not fully understood and is structurally based on a complex with a short model peptide of PFL. Here, we present extensive molecular dynamics simulations in combination with quantum mechanics/molecular mechanics (QM/MM)-based kinetic and thermodynamic reaction evaluations of a more complete activation model comprising the 49 amino acid long C-terminus region of PFL. We reveal the benefits and pitfalls of the current activation model, providing evidence that the bound peptide conformation does not resemble the bound protein–protein complex conformation with PFL, with implications for the activation process. Substitution of the central glycine with (S)- and (R)-alanine showed excellent binding of (R)-alanine over unstable binding of (S)-alanine. Radical stabilization calculations indicate that a higher radical stability of the glycyl radical might not be the sole origin of the evolutionary development of GREs. QM/MM-derived radical formation kinetics further demonstrate feasible activation barriers for both peptide and C-terminus activation, demonstrating why the crystalized model peptide system is an excellent inhibitory system for natural activation. This new evidence supports the theory that GREs converged on glycyl radical formation due to the better conformational accessibility of the glycine radical loop, rather than the highest radical stability of the formed peptide radicals.

## Introduction

Anaerobic glycyl radical enzymes (GREs) are one of the most prominent enzyme families known to catalyze crucial reactions in strict and facultative anaerobes.^1,2^ As part of the human gut microbiome,^3−5^ they also govern key metabolic pathways in their hosts, which link them to several diseases.^6−8^ A detailed understanding of their activation and catalysis, therefore, is of great importance in finding suitable treatments for diseases associated with the intracellular role of GREs. Due to their versatile activity, GREs also have potential applications in biotechnology for producing chemicals in more environmentally sustainable ways.^9^

Although GREs have been extensively studied recently, which led to the discovery of several new members of the enzyme family,^10,11^ the crystal structure of only one activating enzyme has been resolved.^12^ These GRE-activating enzymes (GRE-AEs) are responsible for installing a glycyl radical on the backbone of the corresponding GRE [see refs (2)(13), and (14) for general reviews on the GRE and radical S-adenosylmethionine (rSAM) enzyme mechanisms]. This activation is facilitated by the formation of protein–protein activation complexes whose role in enzyme activation is currently not understood in detail. GRE-AEs are metalloenzymes belonging to a superfamily of radical rSAM enzymes.^15−18^ The formation of the stable complex between the two enzymes and the binding of glycine in the active site of the activase is a prerequisite for the successful activation.^12,17^

**Figure 1:**
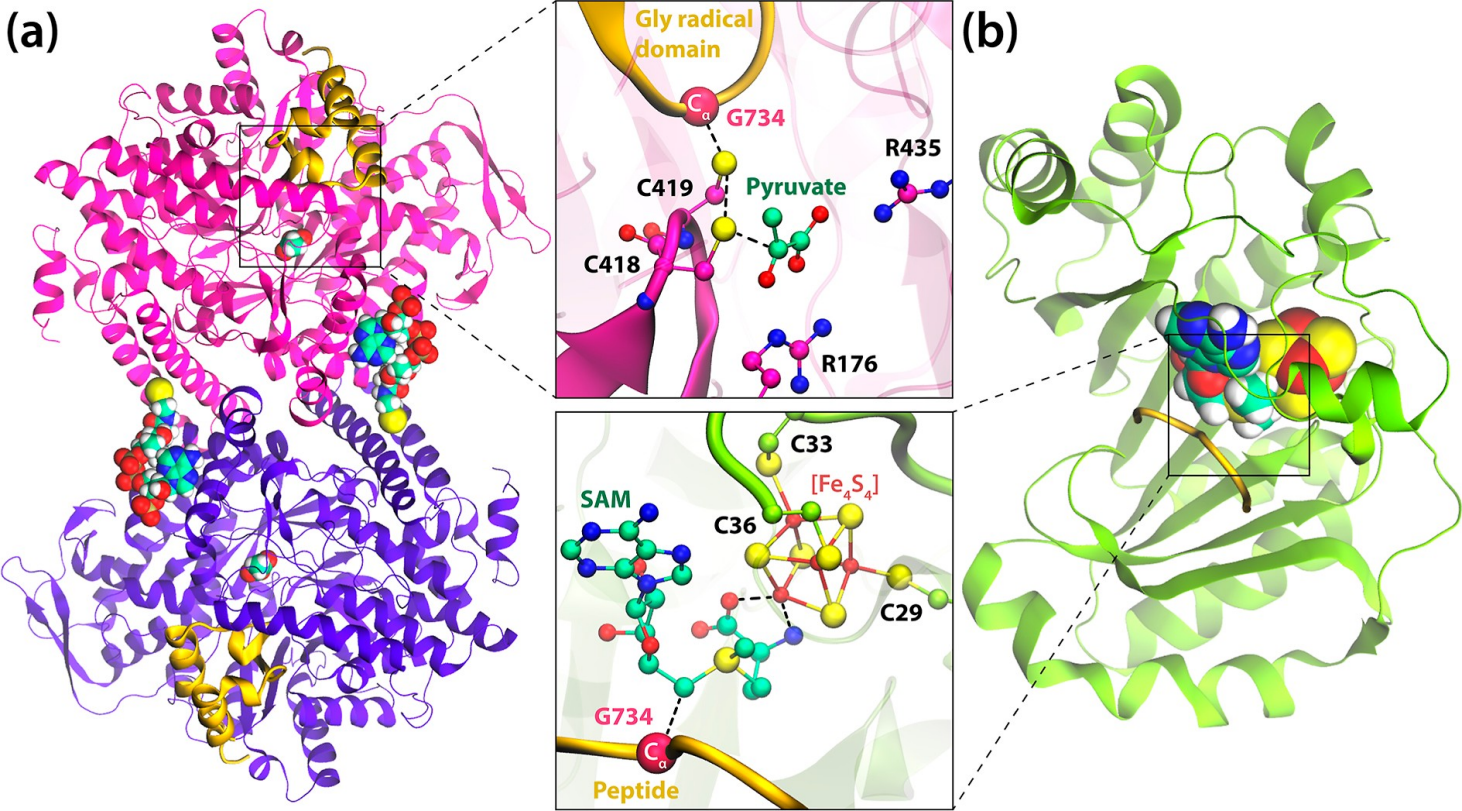
Crystal structure of (a) PFL and (b) PFL-AE with a close view of their active sites. PFL is a homodimer composed of two identical subunits: one depicted as magenta and the other as purple cartoon representation; substrates pyruvate and CoA are shown in ball and stick representation, and the Gly radical domain and Cys loop are shown as orange and magenta ribbons, respectively. The alpha carbon of Gly734 is depicted as a magenta sphere. The monomeric PFL-activating enzyme (PFL-AE) is depicted as green ribbons with SAM and [Fe_4_S_4_] shown as spheres colored by the element. The model peptide substrate bound to PFL-AE is presented as an orange ribbon with the C_α_ atom of Gly734 shown as a magenta sphere.

The transformations catalyzed by GRE members include a broad range of versatile radical-involving reactions,^19^ such as the dehydration of alcohols like choline and glycerol (C–N and C–O bond breaking) by choline trimethylamine-lyase (CutC)^20−23^ and B_12_-independent glycerol dehydratase (B_12_-iGDH),^24−28^ respectively, and of amino acid precursors such as *trans*-4-hydroxy-l-proline via the elimination of water carried out by newly discovered *trans*-4-hydroxy-l-proline dehydratase (HypD).^29^ Other reactions include the C–S bond cleavage of isethionate to yield sulfite and acetaldehyde carried out by isethionate sulfite-lyase (IseG)^7,30^ and class III ribonucleotide reductases (RNRs) that convert nucleotides to deoxynucleotides.^31^ Particularly interesting is also the decarboxylation (C–C cleavage) of aromatic acetates such as *p*-hydroxyphenylacetate carried out by *p*-hydroxyphenylacetate decarboxylase (HPAD)^32^ and the bond making or breaking of challenging C–C bonds carried out by benzylsuccinate synthase (BssA)^33^ and pyruvate formate-lyase (PFL), respectively.^2^ With the exception of class III RNR, all GREs have a similar three-dimensional tertiary structure of the Gly radical domain positioned at the C-terminus that contains an essential Gly loop buried in the interior of the protein (see Figure 1a).^2^

Recently, it has been demonstrated that conformational changes have a critical role in the catalysis of the GRE family member PFL by providing a suitable open state for the activation in the presence of the activase^34^ and additional fluctuations of the channel to accommodate the substrate exclusively after the chemical modification in the active site.^35,36^ However, the crucial large-scale structural rearrangements of PFL, which would bring the Gly loop from the interior to the surface and facilitate the activation process by the activating enzyme, are still not fully understood, albeit their importance has been postulated before.^12^

PFL is a prototypical GRE that converts pyruvate and coenzyme A (CoA) to formate and acetyl-CoA (Figure 1a).^37,38^ PFL participates in the vital step of the anaerobic glucose metabolism of *Escherichia coli* and other microbes, supplying a source of acetyl-CoA in the Krebs cycle. Its activating enzyme PFL activase (PFL-AE)^39^ has been structurally characterized by Vey et al.^12^ with a homologue of the natural peptide sequence from PFL bound in the active site. PFL-AE has also recently been used to finally experimentally prove the existence of the 5′-deoxyadenosyl radical (5′-dAdo^•^) intermediate in the active site^40^ that had been proposed several decades before.

**Figure 2:**
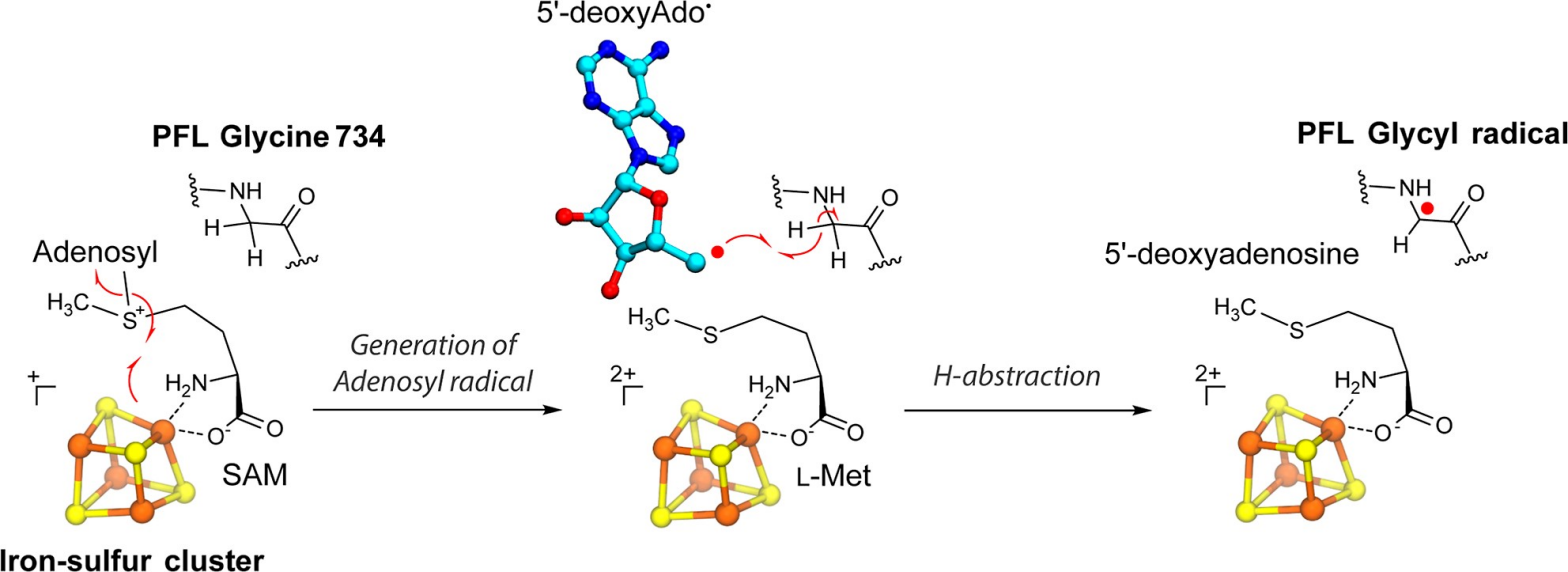
The activation of PFL includes the generation of 5′-dAdo^•^ and subsequent hydrogen abstraction from the PFL protein backbone, catalyzed by PFL-AE.

A crystal structure of PFL-AE reveals the presence of SAM bound to the iron–sulfur [Fe_4_S_4_] cluster in the active site and the Gly-containing peptide that resembles the loop in the Gly radical domain of PFL (Figure 1b).^12^ The activation of PFL catalyzed by PFL-AE is shown in Figure 2. Namely, the generation of the Gly radical is orchestrated by the transfer of a single electron from the reduced [Fe_4_S_4_]^+^ cluster to SAM and the reductive cleavage of the C5′–S bond in SAM generating l-methionine and a central 5′-dAdo^•^ species followed by the hydrogen atom abstraction from the glycine of the bound PFL mediated by the 5′-dAdo^•^ intermediate.^41−43^

The X-ray structure and numerous experimental studies performed on PFL-AE also show that, besides the presence of SAM and the [Fe_4_S_4_] cluster in the active site, the binding of the peptide and a monovalent cation (Na^+^ in the crystal structure) is essential for the initiation of the radical reaction.^44^ The absence of a bound peptide destabilizes SAM and increases fluctuations in certain regions of PFL-AE, which seems to prevent the initial formation of the 5′-dAdo^•^ intermediate.^12^

Further studies including X-ray crystallography by Drennan and co-workers^12^ demonstrate that the activation and cleavage of AdoMet are hindered or even completely abolished in the absence of a bound substrate. This is most likely due to increased flexibility of certain PFL-AE regions involved in the substrate binding, leading to the nonproductive binding of AdoMet, as also indicated by a partially disordered AdoMet in the crystal structure.

Over 2 decades earlier, Knappe and co-workers were first to isolate PFL-AE^45^ and shed light onto general mechanistic and kinetics of the glycine activation in PFL-AE.^42,46^ Utilizing synthetic peptides, including the heptamer later used in the crystallization study and examples where the central Gly is mutated to (*R*)-Ala, they reported the ability of these peptides to function as competitive inhibitors for PFL activation and to directly be activated by PFL-AE, albeit with less activity compared to that of PFL. A peptide comprising natural (*S*)-Ala, on the other hand, totally abolished the interaction with the activase.^42^ However, the exact influence of the peptide binding on the activase catalysis is still not well understood.

More recent studies have focused on the characterization of the PFL repair protein (autonomous glycyl radical cofactor YfiD), which has been described as operating as an independent GRE and whose structure has been the foundation for better understanding the GRE activation and postoxygen exposure repair mechanism. The solved NMR structure of YfiD also has a remarkable primary sequence similarity of 92.2% in the region around Gly734 (see Comment S1 in the Supporting Information for details on the local alignment carried out with LALIGN)^47^ and an identical tertiary structure to the Gly radical domain located at the C-terminus of PFL, which raises hopes that this structure can help in deciphering the protein–protein complex-facilitated activation process in both PFL and GREs in general.^2,48^

In this work, we present detailed new insights into the factors influencing the activation of PFL—as an exemplary GRE-activation process—facilitated via binding to PFL-AE. By extending the current picture that is based on the crystallized short model peptide of PFL bound to PFL-AE and comparing it to the features of a more complete PFL C-terminus model, we highlight characteristics and weaknesses of the current activation model. Applying a broad set of extensive multiscale modeling approaches, including atomistic classical molecular dynamics (MD) simulations, protein–protein docking, and quantum mechanics/molecular mechanics (QM/MM) calculations, we demonstrate how the dynamics and kinetics of activation and the crucial peptide radical formation step are differently influenced in the presence and absence of the model peptide and more realistic longer GRE binding domains. Additionally, we have revisited the question of why GREs have evolved solely around a central Gly instead of other similarly stable peptide radicals.

## Methods

### Model Systems

All enzymatic model systems were derived from a crystal structure containing the PFL-AE monomer in complex with SAM and a model 6-mer peptide [protein data bank (PDB): 3CB8].^12^ The original PDB was modified by assigning the protonation states of titratable residues using the H++ server.^49,50^ Assignments made by the webserver were additionally verified and confirmed by visual inspection of the local environments of the titratable residues. All crystal water molecules, the centrally bound sodium ion, SAM, and [Fe_4_S_4_] were retained, while other cocrystallized species, including formate, were removed from the PDB. Different PFL-AE models representing the enzyme with the peptide containing central glycine, (*S*)- or (*R*)-alanine, and a glycyl or alanyl radical bound in the active site were constructed. The standard acetyl and *N*-methyl amide capping groups were added to the peptide. For comparison, two additional PFL-AE models were constructed, one without the peptide and the other without the peptide and SAM.

### Systems Parameterization

The parameters assigned to standard amino acid residues were taken from the ff14SB force field^51,52^ available within the Amber16 software.^53^ Force field parameters for SAM and the iron–sulfur cluster were obtained from Saez and Vöhringer-Martinez^54,55^ and Carvalho and Swart,^56^ respectively. The parameters for Gly and Ala radicals were derived by adjusting the all-atom optimized potentials for liquid simulation (OPLS-AA) force field parameters initially derived by Komáromi et al.^57^ and Owen et al.,^58^ respectively. Each system was solvated with the extended simple point charge (SPC/E) explicit water model in a truncated octahedron box.^59^ An additional six sodium ions to neutralize the system were added in random positions. The number of water molecules added to the PFL-AE systems was ∼7000 molecules. To compare the bound peptide in the protein to the reference case of a free peptide in water, an additional set of systems where each peptide was placed in a truncated octahedron of ∼3000 water molecules was constructed.

PFL was also studied by introducing a monomeric protein model from the available crystal structure containing bound substrates CoA and pyruvate (PDB: 1H16),^38^ with glycine or a glycyl radical contained in the loop of the Gly radical domain. All crystal water molecules and seven sodium ions present in the PDB were retained. The Mg^2+^ ion was removed and replaced by two Na^+^ ions.^35^ We solvated PFL with ∼23,500 water molecules, neutralizing the system by adding additional eight sodium counter ions. For the nonstandard residues, the missing parameters were derived using the R.E.D. server^60^ and AmberTools17 suite.^61^ These nonstandard residues include the substrates CoA and pyruvate. The CoA parameters were obtained by combining molecular fragments of typical cofactors from the R.E.D. database under the project F-91 provided by Dupradeau.^62^ All phosphate groups of CoA were fully charged. The bonding and nonbonding parameters were taken from the general Amber force field.^63^ The missing charges for pyruvate were obtained by following the standard restrained electrostatic potential (RESP) fitting procedure.^64^ The charges for pyruvate were derived from the electrostatic potential (ESP) calculations at the B3LYP/cc-pVTZ//HF/6-31G(d,p) level of theory combined with an IEFPCM (ε = 4.335) continuum dielectric model. The anionic form of pyruvate was used in parameterization. All QM calculations necessary to derive charges were performed using Gaussian09 software.^65^

### Simulation Details

All systems were treated with periodic boundary conditions. Long-range electrostatic interactions were calculated with the particle mesh Ewald method with a 10 Å nonbonded cutoff for long-range interactions. The temperature was controlled by coupling the system with the Langevin thermostat with the collision frequency set to 2 ps^–1^ in all performed simulations. An integration time step of 2 fs was used, and the SHAKE algorithm was employed to constrain bonds involving hydrogen atoms. Steepest descent minimization was applied to the aqueous solution of the protein–substrate complex (solute) with harmonic positional restraints on solute molecules (2 kcal/mol Å^2^). Heating was performed with continued solute restraints at a constant volume (*NVT*). Thereby, the temperature was increased from 0 to 300 K over 60 ps and kept at that value for another 40 ps. For all systems, 400 ps of constant pressure (*NPT*) dynamics at 300 K was performed, with isotropic position scaling at a pressure of 1 bar and a pressure relaxation time of 0.2 ps using the Berendsen barostat to control the pressure. Finally, an unrestrained *NPT* simulation at 300 K and 1 bar was performed for a duration of 500 ps. The equilibrated systems were subjected to three independent, unrestrained MD production runs (starting from different random atomistic velocity seeds) for 1 μs each, giving rise to an overall simulation time of 3 μs per investigated system. All simulations were propagated at a constant volume and temperature (300 K), saving structures every 10 ps. Simulations of all systems were carried out using the GPU-accelerated *pmemd* module in Amber16 software.^53^ The unrestrained MD simulations of the free peptide in aqueous solution and the PFL monomer were also propagated for a total of 3 μs using the procedure already outlined.

### Protein–Protein Docking

Multiple protein–protein docking protocols have been applied as described in more detail in the Supporting Information (Figure S1). The initial protocols started with attempts to dock the full PFL/PFL-AE model from different structures retrieved from long-timescale MD simulations of individual enzymes, which did not lead to reasonable activation complexes. Based on the observed significant structural changes in the C-terminus region of PFL, subsequent docking protocols involved PFL-AE and the truncated C-terminus of PFL (a total of 49 residues, residues 711–759).

To prepare the system for docking, the crystal structure of PFL-AE was minimized in the presence of the Gly-containing peptide prior to docking, after which the peptide, SAM, the iron–sulfur cluster, ions, and water molecules were removed. Docking was carried out with the ClusPro 2.0 server,^66−69^ keeping the polar hydrogens only and allowing the flexibility of the sidechains.

We specified the attraction and repulsion residues based on the hydrogen bond analysis performed on MD simulations of PFL-AE with the Gly-containing peptide. Namely, while the repulsion residues are usually found deep in the interior of PFL-AE and are not expected to interact with PFL, attractive residues including Asp16 and Asn38, which readily interact with central residues of the PFL loop, are located on the surface of PFL-AE. From the visual inspection of the obtained complex and the comparison with the peptide position in the active site of PFL-AE, the best docking poses were retained from the top docking scores. To obtain the complex of PFL-AE with the (*R*)-Ala-containing C-terminus, we mutated a central Gly734 to (*R*)-Ala. After a short relaxation, the three best docking poses were subject to 10 parallel production MD simulations (100 ns each) using the protocol described earlier. The MD simulations of the isolated C-terminus in water were also carried out for a total of 3 μs using a similar procedure.

### Radical Stabilization Energy Calculations

The calculation of the relative thermodynamic stability of radicals via their radical stabilization energy (RSE), as outlined in the literature for amino acids,^70,71^ radicals in enzymatic catalysis in general,^72^ peptide radicals,^73^ and rSAM enzymes,^74,75^ provides valuable information on the driving factors in enzymatic radical chemistry. To calculate RSEs analogous to the procedure outlined and applied extensively by Zipse and co-workers^72,73,76−78^ directly from molecular simulations, a combination of MD snapshots and QM calculations was applied, as shown in Figure S2 in the Supporting Information. Initial structures were selected based on the free energy landscape created after converting probability data points from the Ramachandran plot of the central residue 734 in the C-terminus bound to PFL-AE or free in the solution. Snapshots representing intermediate states were extracted for further processing. Model dipeptides were constructed by capping a central Gly and (*R*)-Ala with acetyl and *N*-methyl amide groups and optimized in vacuum. The pro-(*S*) hydrogen atom was removed from the initial structure, and the newly constructed radical was further optimized. All RSE calculations were carried out at the G3(MP2)-RAD level of theory.^79,80^ Final energies were corrected to 0 K using B3LYP/6-31G(d) zero-point vibrational energies (ZPVEs) scaled by 0.9806. All QM calculations necessary to evaluate RSEs were performed in Gaussian16 software.^65^

### QM/MM Calculations

QM/MM calculations were carried out within the two-layer ONIOM framework as implemented in the Gaussian16 program^65,81−83^ on a previously minimized set of snapshots retrieved from extensive MD simulations. In this respect, 6 different initial structures were extracted from each of the peptide- and 10 structures from C-terminus-containing systems [both Gly and (*R*)-Ala] bound to PFL-AE. While a great portion of the system was treated classically, the QM region was composed of SAM, the central Gly or (*R*)-Ala residue, and the relevant backbone atoms of the neighboring Ser733 and Tyr735 from the peptide and C-terminus (see Figure S3 in the Supporting Information for a detailed setup). This selection ensured that all residues with direct influence on the radical hydrogen transfer reaction are treated at a QM level adequate for organic radical reactions, while the remaining enzyme including the redox-active [Fe_4_S_4_] cluster (relevant for preceding reaction steps) is treated with classical mechanics. The total charge of the QM region was −1, with a doublet multiplicity specified in all calculations. To prepare models for QM/MM calculations, the closest 100 water molecules surrounding the QM region were retained. All residues within 4 Å of the QM zone were allowed to move freely in the QM/MM optimizations, while the remaining external residues were fixed to provide rigidity to the system. Structure preparation has been performed with the TAO (a toolkit to assist ONIOM calculations) package.^84^ Starting from the substrate geometry with the S–C bond cleaved in SAM, the reaction path was explored by performing potential energy surface (PES) scans, followed by geometry optimizations. The PES scans were performed by decreasing the distance between the C5′ atom of 5′-dAdo^•^ and pro-(*S*) hydrogen of residue 734. At each point of the scan, a constrained geometry optimization was performed. Structures generated from the scans approximated the path of the reaction. The structure with the highest energy along the selected reaction coordinate can be considered as an initial guess of the transition state (TS) geometry and was used to obtain the TS of the examined coordinate. The last structure from the scan was optimized to find the geometry of a new intermediate. The TAO package was used to extract desired geometries from PES scans. Several functionals have been previously tested and reported suitable for accurate description of a similar radical-involving hydrogen abstraction in coenzyme B_12_-dependent enzymes. Following the methodology developed by Wick and Smith,^85^ structures were optimized employing the mechanical embedding at the ONIOM[TPSS/def2-SVP:ff14SB] level of theory, whereby the single-point calculations were performed on the optimized geometries using the electrostatic embedding implementation at the ONIOM[TPSS/def2-TZVP:ff14SB] level, including Grimme’s D3 dispersion correction.^86^ The initial mechanical embedding step ensured convergence and structural accuracy at reasonable computational costs. To validate all optimized geometries, frequency analyses were performed using the same level of theory as that used for the geometry optimization. Final energies were corrected by unscaled ZPVEs and plotted using a reaction coordinate defined in Figure S4 of the Supporting Information.

### Analysis

All trajectories obtained from the MD simulations were subsequently processed and analyzed using the *cpptraj* module of the Amber16 program.^53^ All structures were visualized using VMD 1.9.3.^87^

## Results and Discussion

### PFL-AE-Peptide Binding Model

**Figure 3:**
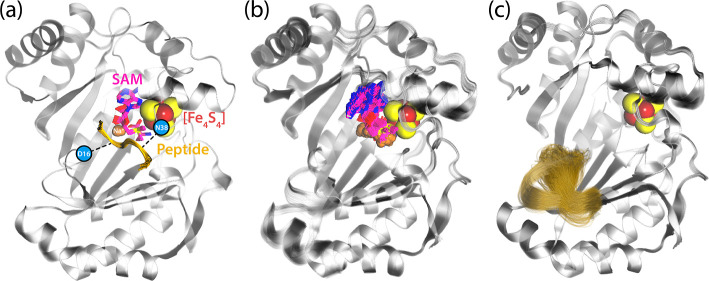
Structures of PFL-AE with (a) and without (b,c) the bound model peptide created by aligning MD snapshots from 3 μs MD simulations. The [Fe_4_S_4_] clusters are shown as spheres, and the interaction of the peptide with anchoring residues Asp16 and Asn38 is highlighted with blue circles and black dashed lines. With SAM and the peptide bound (a), the enzyme shows good structural integrity. Without the peptide (b), SAM shows weak binding and high flexibility. In the case without peptide or SAM bound (c), PFL-AE additionally shows significantly increased flexibility in the peptide-binding region.

First, we investigated the effects of binding of the model peptide (from the published PFL-AE crystal structure) and SAM as cosubstrates to the structural integrity and dynamics of the PFL-AE system to test and explain experimental evidence that peptide binding is essential for the initial formation of the activated SAM (dAdo) radical intermediate.^12^ From a series of microsecond MD simulations of PFL-AE with and without bound model peptide and SAM, clear differences in the dynamic flexibility of the systems can be observed [see Figure 3 and root-mean-square deviation (RMSD) analysis in Figure S5 of the Supporting Information]. Indeed, the enzyme and the active site with bound peptide appear much better stabilized. On the other hand, analysis of the residual root-mean-square fluctuations (RMSF) shows significantly higher fluctuations in the absence of the peptide, especially in the region between residues 10 and 20, as shown in Figures 3c and S6 in the Supporting Information. This region is engaged in the peptide binding via interactions between Gly734 and the anchor residue Asp16, as already explained by Drennan and co-workers.^12^ In their study, it was experimentally shown by X-ray crystallography that this region has increased mobility and thus a distorted structure in the absence of the peptide.

Additionally, we observed a significant mobility of residue Asn38 in the absence of the peptide in all performed simulations. While Asn38 plays an important role in stabilizing the peptide through hydrogen bonds with Gly734 and Ala736, the affinity toward interacting with the central metal cluster increases in the absence of the peptide. From the inspection of the histograms created by collecting the distance data between Asn38 and [Fe_4_S_4_] during MD simulations, we observed that Asn38 is more likely to reside very close to the cluster forming a H–bond with the sulfur atoms from the cluster in the absence of the peptide, as shown in Figure S7 of the Supporting Information. This interaction might play a significant role in influencing the symmetry and redox potential of the cluster and thus impacts the activity of the enzyme, as reported earlier for H–S hydrogen bonding to FeS clusters in other examples.^88,89^

Additionally, the absence of the binding peptide also influences SAM binding, as can be seen from Figure 3b (see Figure S8 of the Supporting Information for further analysis). The bound peptide stabilizes SAM binding, which itself interacts with the unique iron from the [Fe_4_S_4_] cluster through one carboxylate oxygen and the nitrogen of the methionine moiety, while the other carboxylate oxygen of SAM coordinates a Na^+^ ion in the active site. This structured binding is maintained during most simulations with bound peptide over long periods (see Figure S9 of the Supporting Information).

In the absence of the peptide, partial unbinding of SAM is observed in all cases after varying simulation time, as shown in Figure S8 in the Supporting Information. Interestingly, upon the unbinding of SAM, the Na^+^ ion in the active site changes its position and becomes more exposed to the solvent and thus less tightly bound in the active site (see Figure 3b). The cleavage of the crucial interactions with the Na^+^ ion, and its dissociation from the active site, destabilizes the protein interior and introduces additional fluctuations in multiple regions, mainly including those surrounding the metal cluster (residues 130–145, around residues 190 and 205–215) and residues 25–40 which contain three cysteines that coordinate the [Fe_4_S_4_] cluster (see Figures 3b–c and S9 of the Supporting Information). These unbinding and resulting conformational changes could impact the enzyme activity since the correct positioning of the monovalent cations in the active site is known to highly enhance the PFL-AE catalytic efficiency as already highlighted by Shisler et al.^44^

### Comparison of the Peptide Loop Bound to PFL-AE and in PFL

Foreshadowed by the fact that the model peptide cannot reveal how PFL interacts with PFL-AE during activation, we compare the activation of this model peptide with a model of the longer C-terminus of PFL later in this work. Before that, however, it is necessary to understand the conformational and dynamic differences of the PFL binding loop as present in PFL and when bound to PFL-AE.

**Figure 4:**
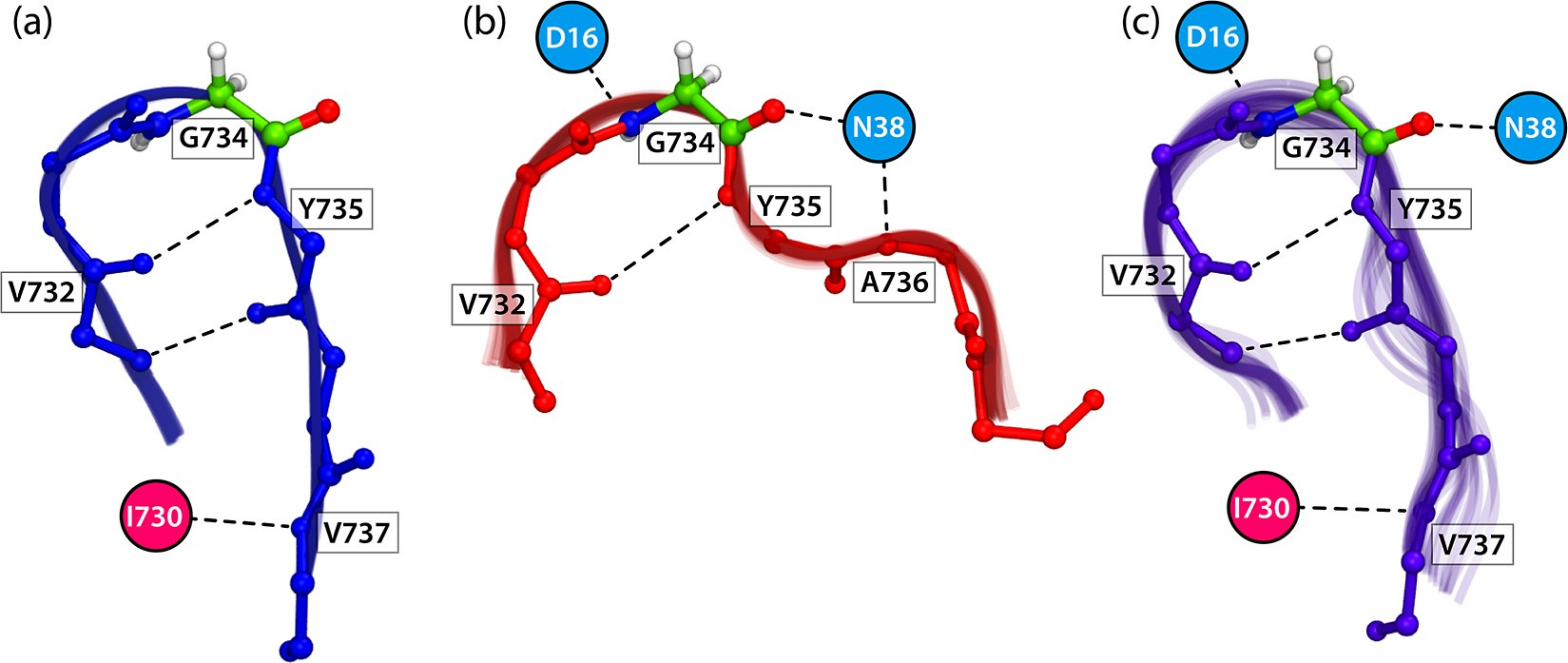
Backbone structures and key hydrogen bonding of the (a) Gly734-containing loop in PFL, (b) peptide, and (c) C-terminus model bound to PFL-AE obtained by aligning the collection of snapshots from 3 μs MD simulations for each system. The loop of PFL exists in the U-conformation (blue), while the bound peptide in PFL-AE adopts the W-conformation (red). The bound C-terminus loop (purple) maintains similar conformation to the Gly-containing loop in PFL. The conformation of the central Gly734 residue (green) remains similar in all cases. The key hydrogen bonding interactions with PFL and PFL-AE are highlighted with red and blue circles, respectively.

In comparison to the structural features of bound peptide in the crystal structure of PFL-AE, the same amino acid sequence in the C-terminus region of PFL shows significantly different conformational behavior and hydrogen bonding patterns, as shown in Figure 4. While the model peptide demonstrates an overall W-shape binding conformation, the analogous Gly-containing binding motif in PFL adopts a compact U-shaped conformation (see Figures 4a–b and S10 in the Supporting Information).

In the U-conformation, amino acids are connected via hydrogen bonds, forming a stable two-stranded antiparallel β-sheet with Gly734 located at the turn of the β-finger motif and deeply buried in the active site of PFL (see Figure 4a). Such a position protects the radical intermediate formed during the activation from undesired side reactions and quenching. Despite the fact that most of the interactions between the Gly loop and the rest of PFL include hydrophobic contacts, the hydrogen bond analysis shows that the most frequent H-bonds observed from MD simulations mainly contribute to the stability of the characteristic β-turn (see Figure S11 and Table S1 of the Supporting Information for details).

On the other hand, the model peptide binds to PFL-AE in a more extended W-conformation, as shown in Figure 4b. This arrangement guarantees multiple contacts with the activase and correct positioning of Gly734 near SAM in the active site, which is a prerequisite for the H-abstraction and activation. The bound W-conformation also demonstrates a significantly different hydrogen bonding pattern when bound to PFL-AE in comparison to the U-fold in PFL, as shown in Figure S12 and Table S2 in the Supporting Information. Specifically, the most frequent H-bonds with PFL-AE observed from MD simulations include the interaction of the central residue 734 with Asp16 (∼80%) and Asn38 (∼40%) and peptide residue Ala736 with Asn38 (∼60%).

The stability of the model peptide in complex with PFL-AE has been analyzed by monitoring the RMSD of the peptide backbone in MD simulations during which the peptide remained tightly bound to the activase. Furthermore, the lowest RMSF were found for the central residues in the peptide, especially residue 734, indicating additional stabilization in the bound state, which is crucial for the initial hydrogen transfer (see Figure S13 in Supporting Information for details).

Analyzing additional simulations incorporating the activated glycyl radical in PFL reveals further differences. Although the flexibility of the Gly loop relaxes shortly after the equilibration and remains stable in the case of the inactive PFL, increased fluctuations are obtained for the loop containing the Gly radical (see Figure S14 in the Supporting Information). The higher flexibility can be explained by the capability of the planar Gly radical to reach the catalytic cysteines in the activated PFL, which is important for the initial hydrogen transfer from Cys418 to Gly^•^ through Cys419. This further allows the generation of a thiyl radical that triggers the catalysis in PFL by breaking pyruvate to a formate radical and an acetyl group.^35,37^

**Figure 5:**
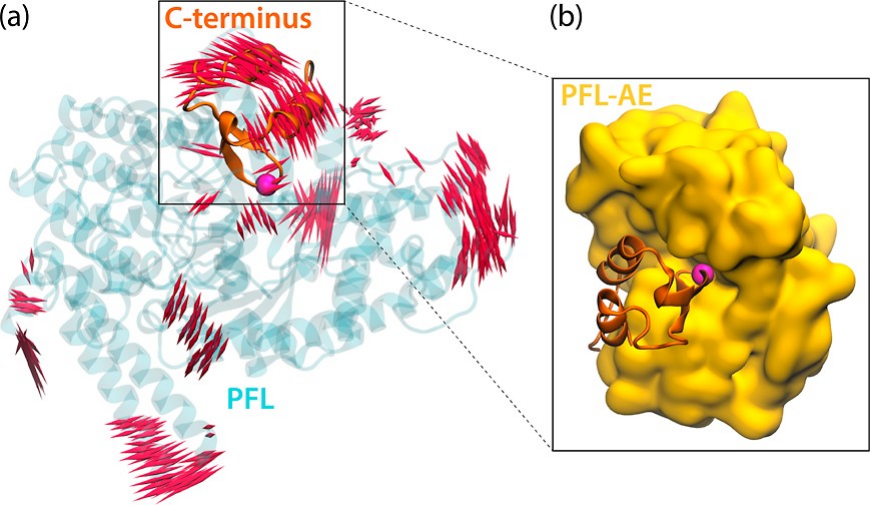
(a) Displacement vectors longer than 2.5 Å for the fourth mode(PC4) shown as red porcupines and drawn in both directions calculated from 3 μs MD simulations of PFL (blue transparent ribbons) containing the nonradical Gly radical domain (shown as orange ribbons). (b) Representative docking pose of the C-terminus of PFL bound to PFL-AE (yellow surface). The alpha carbon atom of Gly734 is shown as a magenta sphere.

The flexibility of the C-terminus region of PFL that is deemed to be crucial for the formation of protein–protein interactions (PPIs) for activation has further been analyzed from 3 μs MD simulations demonstrating significant conformational changes, as shown in Figure 5a based on a principal component analysis (PCA). This conformational change associated with the Gly loop and the two helices covering the active site partially expose Gly734 from its buried state to a more surface-exposed location that is potentially accessible to binding by PFL-AE. However, the complete exposure of the Gly loop, which is necessary for successful binding, requires more drastic conformational changes and has not been observed in any of the performed MD simulations.

As this means that protein–protein docking of the full PFL-PFL-AE system from these structures cannot result in complexes close to possible activation complexes, we extracted multiple solvent exposed structures of the crucial C-terminus region to investigate its binding to PFL-AE. The resulting complexes are shown in Figures 5b and S1 of the Supporting Information.

### The PFL-AE-C-Terminus Binding Model

Long timescale simulations of the docked C-terminus in PFL-AE revealed PFL-AE dynamics with significant differences to the small peptide binding in the crystal structure. We observed stable complex formation between the C-terminus and PFL-AE, resulting in reduced flexibility associated with the backbone of the activase (see Figures S15 and S16 in the Supporting Information). The presence of the C-terminus also stabilized critical protein regions near the binding site and other substrates in the active site (see Figures S17 and S18 of the Supporting Information) which is crucial for the catalysis. However, compared to the model peptide bound to PFL-AE, the bound C-terminus demonstrates slightly higher flexibility.

The results indicate that the docked poses of the C-terminus are indeed suitable substrates for PFL-AE since the Gly loop remains bound in the active site closely interacting with SAM in most of the performed simulations, significantly stabilizing the activating enzyme.

To compare the overall structural and dynamic differences of the C-terminus and model peptide binding to PFL-AE, we performed PCA on MD simulations of those complexes and free substrates in solution (see Figure S19 of the Supporting Information). While isolated peptides in aqueous solution demonstrate higher conformational diversity, the peptide bound to PFL-AE displays minor flexibility and limited conformational freedom, remaining mostly in the W-conformation. However, both free and bound C-termini exhibit more rigid structures where the Gly loop remains mostly in the U-conformation, sharing similar structural features with the conformation of the Gly loop in the interior of PFL.

**Figure 6:**
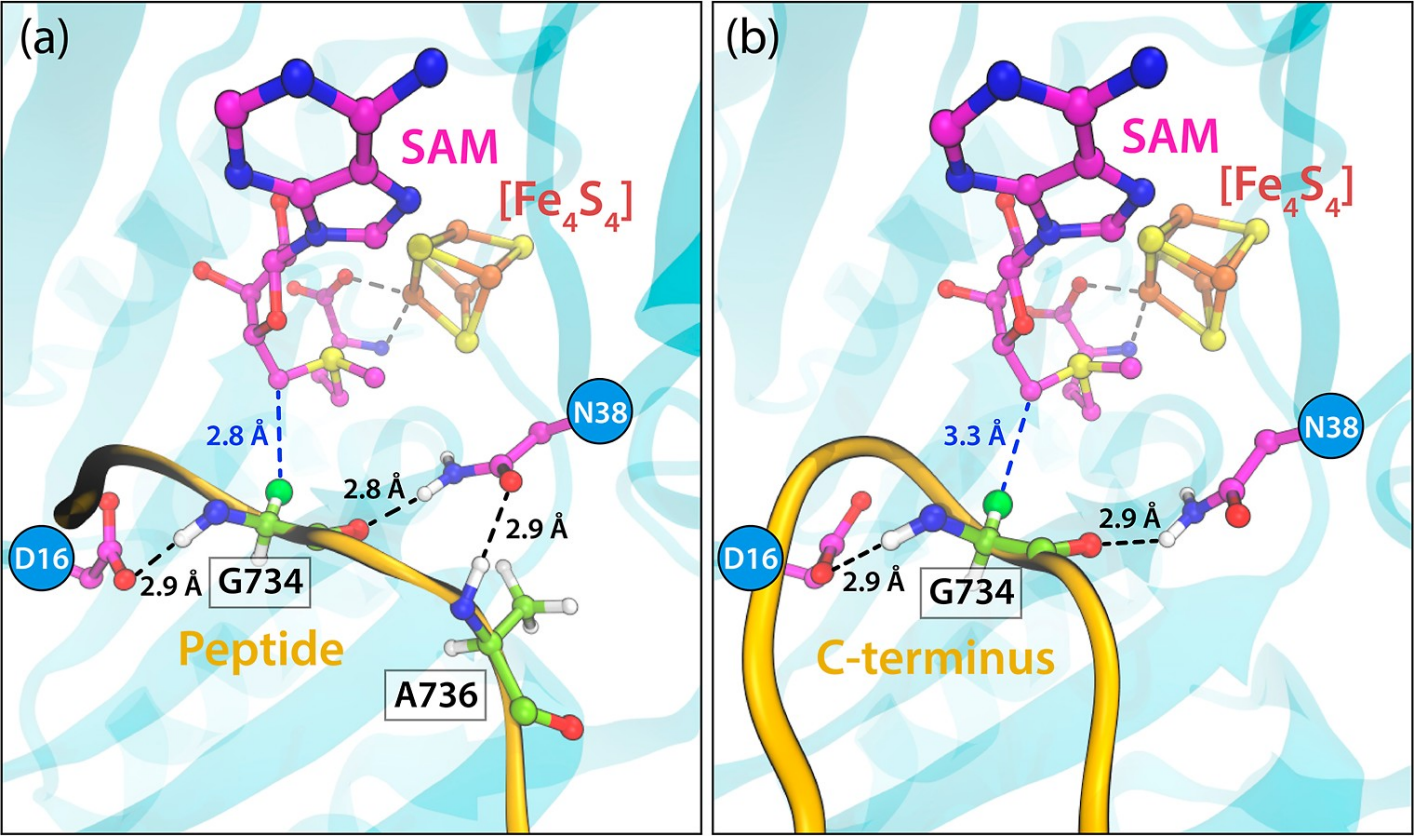
Representative structures extracted from 3 μs MD simulations showing the (a) peptide and (b) C-terminus bound to PFL-AE. Crucial frequent interactions with Asp16 and Asn38 are highlighted in black and with SAM in blue dashed lines.

Regarding the anchoring of the C-terminus in the active site, the H-bond analysis (see Figure S20 and Table S3 of the Supporting Information) shows up to 30 and 10% less persistent interactions of the central residue 734 with Asp16 and Asn38, respectively, compared to those of the bound short peptide. Moreover, due to the engagement of most loop residues in stabilizing the U-conformation, we rarely observe the evidence for a H-bond between Ala736 from the C-terminus and Asn38 from the activase (see Figure 6 for details on representative MD snapshots featuring common contacts with PFL-AE). A distance probability distribution analysis also revealed that while the hydrogen bonding network of the central Gly appears to be highly similar for the bound peptide and the C-terminus, the distance between the C_α_ of the central residue and the C5′ of SAM was found to be around 0.5 Å longer in the C-terminus simulations (see Figure S21 in the Supporting Information).

Finally, although the docked C-terminus stays bound to the activase throughout the entire simulation time, we observed a significantly increased flexibility of the U-loop compared with that of the bound peptide (see Figure 4c showing the collection of superimposed conformations of the more flexible C-terminus loop bound to PFL-AE). This implies that irrespective of the larger binding interface, the central glycyl radical loop region of the C-terminus binds less tightly to PFL-AE than the model peptide. However, it remains unclear at this point to which extent additional, more distal interactions in the full PPI complex are likely to compensate for the lower affinity of the central loop. This would further indicate that distal contributions play a more important role in activation than observable from the available experimental and theoretical results until now. Overall, the binding affinity between PFL and PFL-AE is on the weaker end of biological interactions (a *K*_D_ of 1.1 ± 0.2 μM) and thought to be rate limited by significant conformational changes in both enzymes, as reported by Crain and Broderick.^90^

### Binding Alternative Sequences: Alanine Mutants

A key question regarding GREs in general is why these enzymes exclusively evolved to feature Gly radicals. It has been discussed that the loop formation and the stability of the “inexpensive” glycyl radical might have been evolutionary drivers.^2,73^ However, alanyl radicals have been established as being able to work similarly adequately.^42^ Thus, we investigated Ala activation in this study as well and have first investigated the binding of (*R*)-Ala and (*S*)-Ala peptide variants.

Simulations of PFL-AE with an (*S*)-Ala734 mutant of the crystallized model peptide showed significantly higher fluctuations and unfavorable binding (see Supporting Information Figure S12 for details). The increased flexibility can mainly be attributed to the steric effect of the methyl group pointing toward SAM in the active site, also leading to a 20% less frequent occurrence of the key hydrogen bonds anchoring the peptide into the active site of PFL-AE (see Table S2 in the Supporting Information).

**Figure 7:**
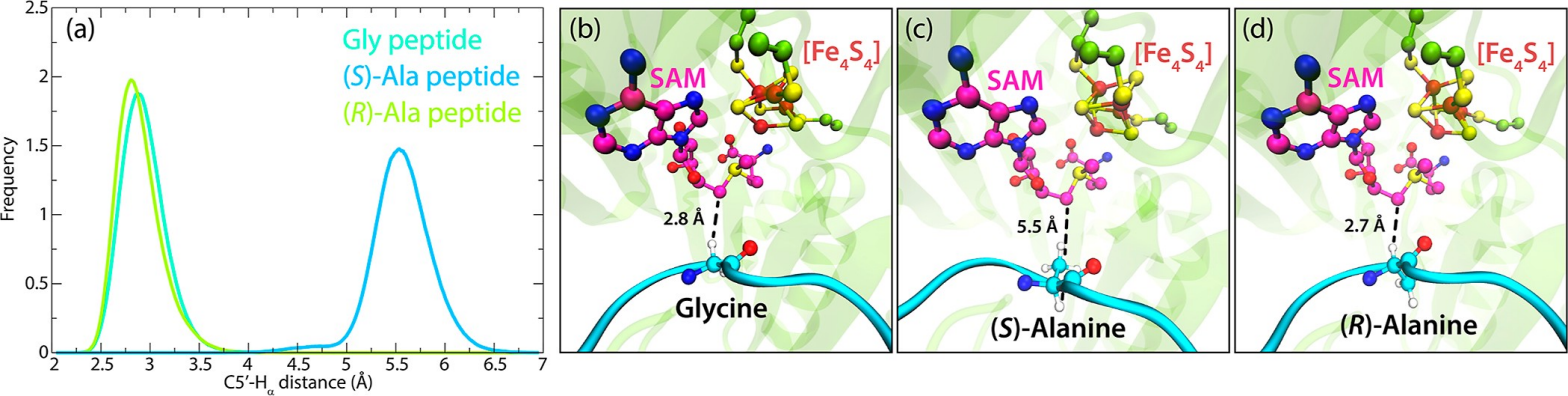
(a) Probability distribution of the distance between C5′ from SAM and H_α_ from the central residue 734 in the model peptide. Representative snapshots of (b) glycine-, (c) (*S*)-alanine-, and (d) (*R*)-alanine-containing peptides bound to PFL-AE taken from MD simulations. The histograms were created by collecting distance data from 3 μs MD simulations of each peptide bound to PFL-AE.

In contrast to the (*S*)-Ala variant, the non-natural (*R*)-Ala variant does not display similar steric clashes and indeed binds almost identically to the Gly variant. Analyzing the C5′-H_α_ distance between SAM and central residue 734 during MD simulations of all three variants also showed significant differences. As shown in Figure 7, peptides containing the central Gly or (*R*)-Ala bind closer to SAM in a favorable position suitable for hydrogen abstraction.

In contrast, the (*S*)-Ala variant depicts a much longer hydrogen transfer distance throughout the whole simulation. This indicates less probable H-transfer since at no time in the simulations a linear hydrogen transfer could occur as demonstrated in the distance probability distribution shown in Figure 7a and the orientation of the C_α_–H_α_ bond vector in (*S*)-Ala that points in the opposite direction to the C5′ atom from SAM (see Figure 7b–d). These results strongly indicate that only Gly and (*R*)-Ala variants bind in a suitable position for the reaction with the adenosyl radical, while the (*S*)-Ala variant binds in a conformation not suitable for direct linear hydrogen transfer and activation.

### Relevance of Radical Stability on PFL Activation

As discussed in the literature, the radical stabilization of the activated Gly radical might play a crucial role in the GRE activation and mechanism.^73^ Better stabilized radicals are longer lived, more likely to “survive” the PPI-mediated activation and less likely to undergo unwanted side reactions. C_α_ peptide backbone radicals are particularly well stabilized by spin delocalization of the planar radical center supported by the captodative effect.^91−94^ Preventing the planarization of the radical center diminishes this effect as we could demonstrate recently for the radical enzyme QueE^74,75^ and earlier for protected amino acids.^70^ Here, we calculated the radical stabilization energies of the different binding conformations of dipeptides and compared them with the RSE values from dipeptides in solution.

**Figure 8:**
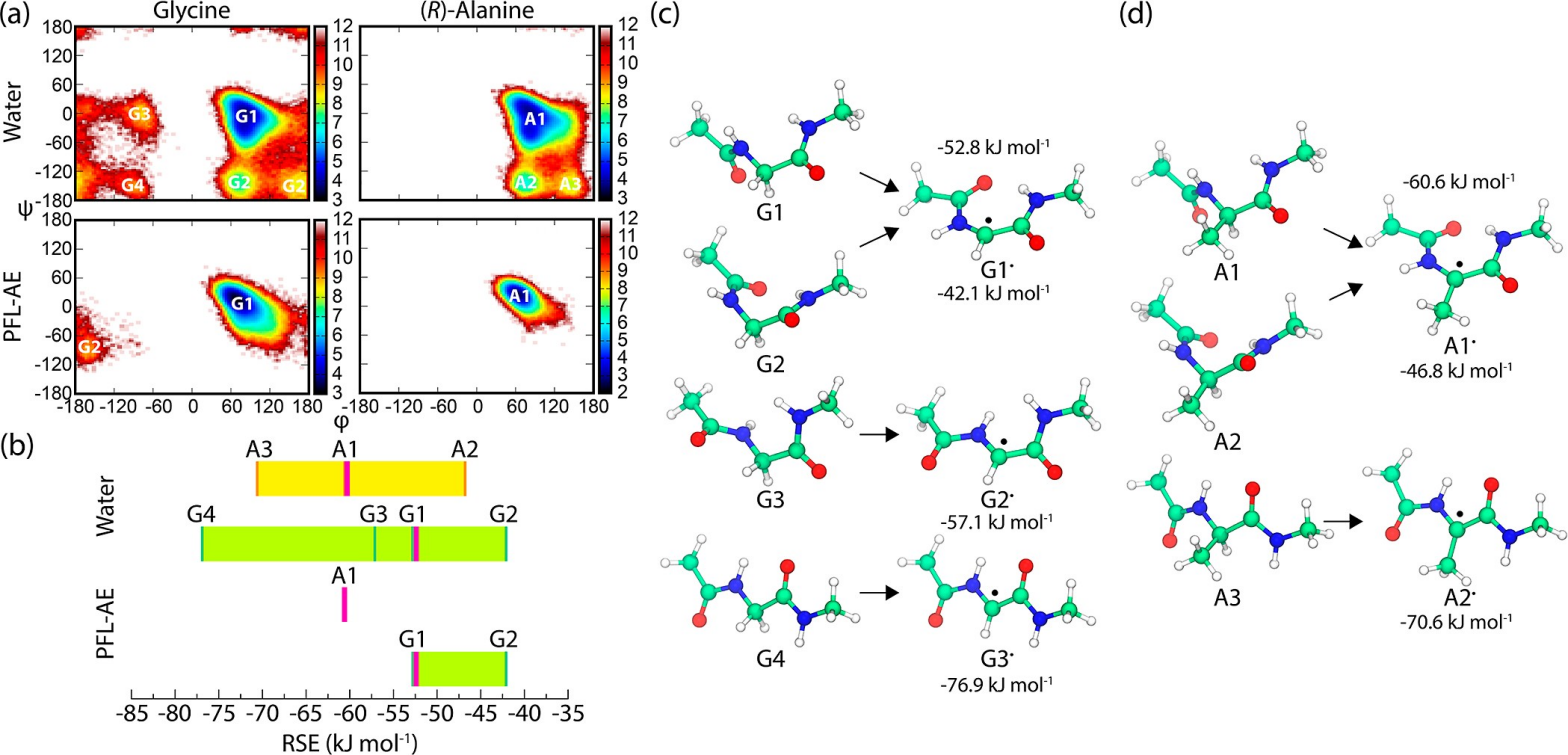
(a) Ramachandran plots (shown as free energy landscapes) of the Gly and (*R*)-Ala residue from MD simulations of the C-terminus in water and bound to PFL-AE. (b) RSE values range (with the Boltzmann average from MD sampling, shown as a bold magenta band) for generating radical species from the obtained conformers in water and bound to PFL-AE. (c) Inspecting the conformation of the central Gly in water and bound to PFL-AE, a total of four conformers were characterized (G1–G4). While conformers G2–G4 occasionally appear in the Gly-containing C-terminus in water, conformer G1 dominates both in water and when bound to PFL-AE. (d) In contrast, three different conformers (A1–A3) were characterized for (*R*)-Ala in water, with conformer A1 prevailing when bound to PFL-AE. The free energy (*k*_B_*T*) heatmap is defined by *W*_i_/*k*_B_*T* = −ln(*N*_i_/*N*_tot_), where *N*_tot_ is the total number of configurations in each system (*N*_tot_ = 300 000 and *T* = 300 K). An equal number of bins (100) has been specified for each dimension. The energies were calculated with the G3(MP2)-RAD method.

RSE values for Gly and (*R*)-Ala dipeptide radicals were calculated by employing high-level QM methods using the representative structures from MD simulations of the C-terminus in solution or bound to PFL-AE. The conformations for these calculations were harvested from free energy landscapes calculated from Ramachandran plots from the respective simulations shown in Figure 8a. The initial Ramachandran diagrams were created by plotting the dihedral angle ψ against φ for the central amino acid. Although Gly and (*R*)-Ala dipeptides can adopt many different conformations in the gas phase,^73^ the conformation of the central residue in the C-terminus is restricted to only a few available conformers due to a structural restraint conferred by the rest of the protein. The dominant conformers G1 and A1 prevail in simulations of both free and bound C-termini, mainly contributing to the Boltzmann average. Due to the interaction of the C-terminus with PFL-AE in the bound state, the Ramachandran space occupied by G1 and A1 is more localized than in water where we found a slightly broader conformational space characterized by a shift in the Ramachandran angles.

The obtained RSE values for Gly and (*R*)-Ala model dipeptides suggest that the most stable glycyl radical G3^•^ is 6.3 kJ mol^–1^ more stable than the corresponding alanyl radical A2^•^. This is mainly due to the unfavorable steric clash between the methyl group and the neighboring peptide moieties in the planar A2^•^ conformation. However, the RSEs calculated for the dominant conformers present in MD simulations of the bound C-terminus suggest that A1^•^ is 7.8 kJ mol^–1^ more stable than G1^•^. This observation is consistent with the additional methyl group stabilization effect for carbon-centered radicals but contrasts with the conformational analysis-based RSEs from solution that support a higher Gly radical stability.^73^ This discrepancy can be explained by the observation that the most stable Gly conformation (G4) is either not accessible or is extremely rare on binding to PFL-AE. Moreover, the formation of the highly stabilized radical G3^•^ from G4 could lead to the inactivation of PFL since such a radical would have difficulty in reacting in further stages of the catalysis. This itself is an intriguing outcome as intuition might suggest that the most stable radical should be formed in PFL-AE in order to prevent its rapid quenching (see Figure 8b–d). Similar conformations G1 and G1^•^ were also found to be dominant in PFL due to the structural restraints of the central glycine residue located in the turn of the beta-hairpin motif in the glycyl radical domain. This leads to identical radical stabilization of the Gly residue in PFL in comparison to that in the PFL-AE-bound case (see Figure S22 in Supporting Information).

The observation that the (*R*)-Ala radicals formed in PFL-AE are more stable than Gly radicals suggests that the radical stability itself cannot explain the existence of radical enzymes that prefer glycine over alanine substrates. Thus, it appears more likely that the evolutionary pressure relies more on the accessibility of the required loop conformation to bind the activating enzyme. This is demonstrated by the less favorable binding of the natural (*S*)-Ala substrates with the α-hydrogen atom pointing away from the hydrogen abstraction direction. This is caused simply by the inaccessibility of the opposite orientation for (*S*)-Ala in this conformation of the loop, which lies in the “forbidden” zone in the Ramachandran space.

### Differences in Glycine Activation by Hydrogen Abstraction

Finally, to evaluate the effects of binding and radical stability on the actual radical formation kinetics, we carried out QM/MM calculations of the energy profile for the initial hydrogen abstraction based on the previously described MD sampling of bound peptides. The starting structures for the QM/MM calculations were chosen from the probability distribution of the C5′–H_α_ distance to represent the distribution peaks and closest contacts.

**Figure 9:**
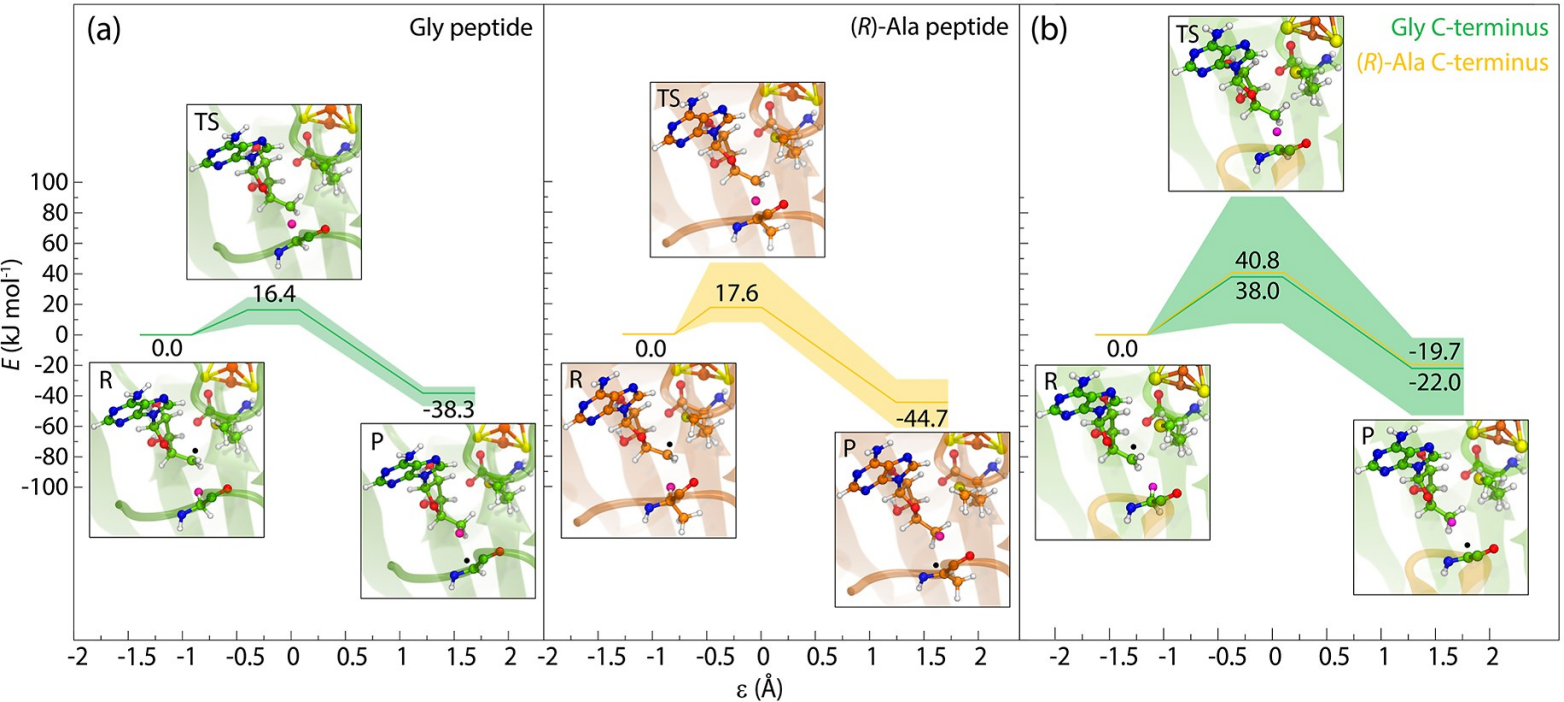
Calculated energy profiles (with lowest, highest, and average values highlighted) for the hydrogen transfer reaction between 5′-dAdo^•^ and Gly (green) and (*R*)-Ala (orange) from the (a) peptide and (b) C-terminus. The hydrogen atom transferred from the substrate to the adenosyl radical is shown as a magenta sphere. The position of the radical in each step is shown as black “•”. The energies were evaluated with ONIOM[TPSS + D3/def2-TZVP:Amber]. In the case of the Gly and (*R*)-Ala peptides bound to PFL-AE, the profiles were calculated using *N* = 6 structures for each peptide variant. The average ± standard deviation (std. dev.) for the TSs were 16.4 ± 6.5 and 17.6 ± 13.4 kJ mol^–1^, while the values for the products were −38.3 ± 4.0 and −44.7 ± 11.9 kJ mol^–1^ for the Gly and (*R*)-Ala peptide, respectively. The number of snapshots used to calculate profiles for the Gly C-terminus bound to PFL-AE was *N* = 10 with an average ± std. dev. of 39.1 ± 21.9 and −24.2 ± 13.9 kJ mol^–1^ for the TS and product, respectively.

The QM/MM calculations summarized in Figure 9a demonstrate relatively broad activation energy distributions and reaction energies. For the bound model peptides, the average barrier for the hydrogen transfer from Gly to 5′-dAdo^•^ is 16.4 ± 6.5 kJ mol^–1^ and 17.6 ± 13.4 kJ mol^–1^ in the case of the (*R*)-Ala-containing peptide. Although the calculated barriers for H abstraction are similar for both Gly and (*R*)-Ala peptides, the newly formed alanyl radical was found to be 6.4 kJ mol^–1^ more stable than the glycyl radical. The choice of the initial MD snapshot used in QM/MM calculations had a minor influence on the resulting energy profiles. The monitored distances *d*_1_ and *d*_2_ and the reaction coordinate ε in each intermediate suggested an early TS for the reaction with both Gly and (*R*)-Ala, as shown in Figure S23 of the Supporting Information.

Calculated energy barriers for the hydrogen transfer between Gly and (*R*)-Ala of the C-terminus and the adenosyl radical were found to be twice as high as those in the reaction with the peptide, which is most likely caused by the increased flexibility of a loosely bound U-loop and a longer distance between the central residue and SAM obtained from MD simulations (see Figures 9b and S24 in the Supporting Information). However, even with barriers of ∼40 ± 20 kJ mol^–1^ for Gly and (*R*)-Ala, they still fall below many hydrogen abstraction energies in radical enzymes, for example, B_12_-dependent diol dehydratase (60–70 kJ mol^–1^),^95^ and lie below many rate-determining energy barriers in the rSAM enzymes’ multistep reactions.^74,96−99^

Our results indicate that competitive binding and/or faster glycine activation of the short peptide sequence could explain the experimentally observed competitive inhibitor role of the model peptide.^42^ This result also does not contradict the results from the study by Knappe and co-workers^42^ that demonstrated the activity of the peptide for the formation of the 5′-dAdo side product, as it might appear at first sight, as this observation can have several reasons. It could, for instance, well be that the preceding electron transfer and 5′-dAdo radical formation kinetics or the dissociation kinetics of the product complexes differ significantly between peptide and PFL binding, influencing the measured overall enzyme kinetics. As the C-terminus binding still misses the full PPIs in the physiological activation complex, this demonstrates once more why a complete PPI model will eventually be needed to fully reveal all driving factors for GRE activation.

## Conclusions

We have investigated the effects of the peptide and C-terminus PFL substrate binding on the stability of PFL-AE and potential implications on the radical formation in PFL by employing atomic MD simulations and QM/MM calculations. From extensive simulations and binding analysis of different variants [Gly and (*S*)- and (*R*)-Ala] of the model 6-mer peptide that has been used for crystallization and mechanistic studies, we demonstrated tight and favorable binding of the Gly and (*R*)-Ala peptide, while the (*S*)-Ala variant showed less favorable binding. This unfavorable binding is mainly driven by (*S*)-Ala not being able to adopt the best conformation for hydrogen transfer and its methyl sidechain creating steric clashes. All peptides significantly stabilized the enzyme structure and the active site of PFL-AE. Significantly increased mobility and weak SAM binding were displayed in their absence. These findings align with the experimental evidence of Knappe and co-workers,^42^ demonstrating the catalytic activity of the Gly and the (*R*)-Ala heptapeptide and the findings of increased disorder of bound SAM in the published crystal structure by Vey et al.^12^

Subsequent calculations of radical stabilization energies and energy barriers for the central hydrogen abstraction showed that the (*R*)-Ala peptide can indeed be activated when binding to PFL-AE, while this is less likely for the proteinogenic (*S*)-Ala variant. Competitively strong binding and easy activation of the small peptide also demonstrate why this peptide has both strong competitive inhibiting effects for PFL^42^ and serves as a perfect template for mechanistic studies.^40,100^

The evaluation of radical stability based on conformational sampling from extensive MD simulations further showed that the radical stability of Gly cannot be the sole reason for its prevalence in enzymes carrying backbone carbon radicals. Indeed, we could see that alanyl radicals are most likely more stable when activated in PFL-AE and that the most stable Gly radical in solution is not formed in the enzyme. While the activation potential of the (*R*)*-*Ala peptide has been shown by Knappe earlier,^42^ our results in particular highlight that this is predominantly due to conformational accessibility within the loop structure, albeit the Ala radicals also being slightly more stable compared to their Gly counterparts. This guiding principle could also be exploited in future experimental mechanistic studies or even as a design principle for future artificial enzyme catalysis.

This observation particularly adds substance to the discussion on the evolutionary development of GREs that these structures are more likely driven by conformational requirements for the radical-carrying peptide loop than by radical stability. It also adds to understanding the effects of dynamic constraints within the enzymatic complexes on radical stability and reactivity that are not available from in vacuo evaluations. Due to selective binding of peptide conformers and structural restraints in the binding complex, the most stable radical conformations in solution (or gas phase) are not dominant when the peptide is enzyme bound. Furthermore, the stabilization of the C_α_ peptide radical by spin delocalization and supported by the captodative effect^91−94^ can also be hindered in the bound state due to imperfect planarization of the product radical (see also refs (70)(74), and (75)). These effects cannot be seen from static computational evaluations and highlight the importance of the presented combination of long timescale MD simulations with QM evaluations.

The investigations of the analogous peptide loop in PFL demonstrated that the same peptide sequence shows a remarkably different conformational ensemble in PFL compared to that of the model peptide, raising questions of whether the model peptide can fully represent the natural PFL activation mechanism. Long timescale MD simulations delivered partial exposure of the PFL C-terminus binding region to PFL-AE that we have used to create a new PFL activation complex model.

This C-terminus peptide sequence demonstrated proper binding to PFL-AE where the loop confirmation retained the shape of the loop in PFL, in contrast to the small model peptide. This indicates that the model peptide indeed demonstrates an artificially strong interaction, different to the natural activation, where the loop itself is bound more loosely, and the whole protein–protein complex formation relies on additional support via distal interactions, as also postulated by Drennan and co-workers.^12^ A final evaluation of the activation energy barrier for the central hydrogen abstraction reaction confirmed a binding complex prearranged and ready for activation, albeit its activation barrier was higher than that of the model peptide.

This study clearly shows how a C-terminus model of PFL is an adequate model for GRE activation by PFL-AE and highlights the limits of the small peptide model. However, a clear answer to the concrete activation picture and all driving forces can only be given by the not yet accessible full PFL-PFL-AE PPI model that will also be able to explain the large conformational changes of PFL needed to undergo activation. This demonstrates once more the fascinating complexity of reactions facilitated by complex and dynamic PPIs.

## Supporting Information Available

Local sequence similarity analysis between the C-terminus of PFL and YfiD; detailed description of QM/MM setups; docking protocols; additional analysis from MD simulations including PCA, RMSD, hydrogen bonding, and distance analysis; structural coordinates; QM and QM/MM input and archive files; force field parameters and full MD inputs; and details on protein–protein docking results are freely available on Figshare: https://doi.org/10.6084/m9.figshare.17029778.v4 (PDF)
