# Supplementary material for: Activation of Glycyl Radical Enzymes—Multiscale Modeling Insights into Catalysis and Radical Control in a Pyruvate Formate-Lyase-Activating Enzyme
Source: J Chem Inf Model. 2022 Jun 30;62(14):3401–14. doi: 10.1021/acs.jcim.2c00362 (PMC9326890; doi:10.1021/acs.jcim.2c00362)
Supplement: Supplementary file 1 — ci2c00362_si_001.pdf [file ci2c00362_si_001.pdf]

Supporting Information

The Activation of Glycyl Radical Enzymes – Multiscale Modelling Insights into Catalysis and Radical Control in Pyruvate Formate-Lyase Activating Enzyme

Marko Hanževački,[a] Anna K. Croft,[a,*] Christof M. Jäger[a,*]

[a]Department of Chemical and Environmental Engineering, University of Nottingham, Nottingham, NG7 2RD, United Kingdom

*E-mail: anna.croft@nottingham.ac.uk, christof.jaeger@nottingham.ac.uk

Table of contents…………………………………………………………………………...S1

Comment S1 The local sequence similarity analysis between C-terminus of PFL and YfiD……..……S3

Figure S1 Protein-protein docking protocol……………………………………………………S4

Figure S2 Protocol for RSE calculations………………………………………………………...S5

Figure S3 QM/MM calculations setup……………………………………………………..…S6

Figure S4 Definition of reaction coordinate for H-atom transfer……………………………...S6

Figure S5 RMSD of protein backbone calculated from the initial crystal structure of PFL-AE……….S7

Figure S6 RMSF of protein backbone calculated from the initial crystal structure of PFL-AE………..S7

Figure S7 Probability distribution of distance between amide N-atom from Asn38 and S-atom from the iron-sulfur cluster...S7

Figure S8 RMSD of SAM non-hydrogen atoms calculated from the initial crystal structure containing PFL-AE……………………………………………………………………………………….…S8

Figure S9 RMSD of $[Fe_4S_4]$ cluster calculated from the initial crystal structure containing PFL-AE....S8

Figure S10 Average structures of non-radical- and radical-containing peptide bound to PFL-AE and Gly radical domain in PFL………………………………………………………..………………...S9

Figure S11 Average structures of Gly- and Gly radical-containing Gly radical domain in PFL showing important interactions……………………………………………………………………..........S10

Table S1 Hydrogen bonds between Gly radical domain and the rest of PFL….….……..……………S10

Figure S12 Average structures of non-radical- and radical-containing peptide bound to PFL-AE showing important interactions……………….…………………………………………………...S12

Table S2 Hydrogen bonds between model peptide and PFL-AE…………………………………….…S12

Figure S13 RMSD and RMSF of peptide backbone calculated from the initial crystal structure of the peptide in complex with PFL-AE….…………………………………………………….…...S15

Figure S14 RMSD and RMSF of Gly loop backbone calculated from the initial crystal structure of PFL……………………………………………………………………………………………..S15

Figure S15 RMSD of PFL-AE backbone calculated from the initial crystal structure of PFL-AE…...S16

Figure S16 RMSF of PFL-AE backbone calculated from the initial crystal structure of PFL-AE…...S16

Figure S17 RMSD of SAM non-hydrogen atoms calculated from the initial crystal structure containing PFL-AE in complex with C-terminus…………………………………………………………..S17

Figure S18 RMSD of $[Fe_4S_4]$ cluster calculated from the initial crystal structure containing PFL-AE in complex with C-terminus………………………………………………………...……...S17

Figure S19 Free energy landscape along PC1 and PC2 of peptide, C-terminus loop residues and PFL loop residues in water and bound to PFL-AE………………………………………………..………S18

Figure S20 Representative structure of C-terminus with central (a) Gly and (b) (*R*)-Ala bound to PFL-AE extracted from 3 μs MD simulations………………………………………………...……S19

Table S3 Hydrogen bonds between C-terminus of PFL and PFL-AE………………………..……S20

Figure S21 Probability distribution of interactions between peptide or C-terminus and PFL-AE……S21

Figure S22 Ramachandran plots of the non-radical and radical Gly734 residue from MD simulations of PFL shown as free energy landscape. Cartoon representation of a β-hairpin motif with glycine and glycyl radical located in the turn. Optimised dipeptide models of the dominant conformer G1 and G1• used in the radical stabilisation energy calculations……………………………………….………S22

Figure S23 Values of the reaction coordinate and distances d_1 and d_2 in MD snapshots and QM/MM optimised structures during the H-atom transfer between 5'-dAdo• and peptide containing central Gly or (*R*)-Ala……………………………………………………………………….…………S23

Figure S24 Values of the reaction coordinate and distances d_1 and d_2 in MD snapshots and QM/MM optimised structures during the H-atom transfer between 5'-dAdo• and C-terminus containing central Gly or (*R*)-Ala………………………………………………………………………….…….S23

Table S4 The sum of G3(MP2)-RAD and scaled zero-point energies for molecular species used in RSE calculations……………………………………………………………………………..S24

Table S5 The sum of ONIOM and zero-point energies for reactants, transition states and products calculated at the ONIOM[TPSS+D3/def2-TZVP:Amber] level of theory. The lowest harmonic frequencies ω_1 obtained from vibrational analysis of transition states with the ONIOM[TPSS+D3/def2-SVP:Amber] method……………………………………………………………………………S24

```
grcA (PFL) 127 aa in 1 sequence
pflB (YfiD) 127 aa in 1 sequence

Waterman-Eggert score: 324; 104.4 bits; E (1) < 6.2e-28
76.6% identity (92.2% similar) in 64 aa overlap (64-127:64-127)
            70        80        90       100       110       120
grcA   EVRVEGGQHLNVNVLRRETLEDAVKHPEKYPQLTIRVSGYAVRFNSLTPEQQRDVIARTF
       :. .::::::::::. :: : ::...:::::::::::::::::::::: :::.:::.:::
pflB   EASIEGGQHLNVNVMNREMLLDAMENPEKYPQLTIRVSGYAVRFNSLTKEQQQDVITRTF
            70        80        90       100       110       120
grcA   TESL
       :.:.
pflB   TQSM

Waterman-Eggert score: 36; 15.2 bits; E (1) < 0.35
24.1% identity (58.6% similar) in 29 aa overlap (60-87:91-119)
      60         70        80
grcA   EVKPEVRVE-GGQHLNVNVLRRETLEDAV
       :  :.. .. .:  .  : : .:  .:..
pflB   EKYPQLTIRVSGYAVRFNSLTKEQQQDVI
              100       110

Waterman-Eggert score: 30; 13.3 bits; E (1) < 0.79
38.5% identity (69.2% similar) in 13 aa overlap (12-24:74-86)
              20
grcA   NDDLLNSFWLLDS
       : ...:   :::.
pflB   NVNVMNREMLLDA
            80
```

Comment S1 The local sequence similarity analysis between C-terminus of PFL and YfiD.

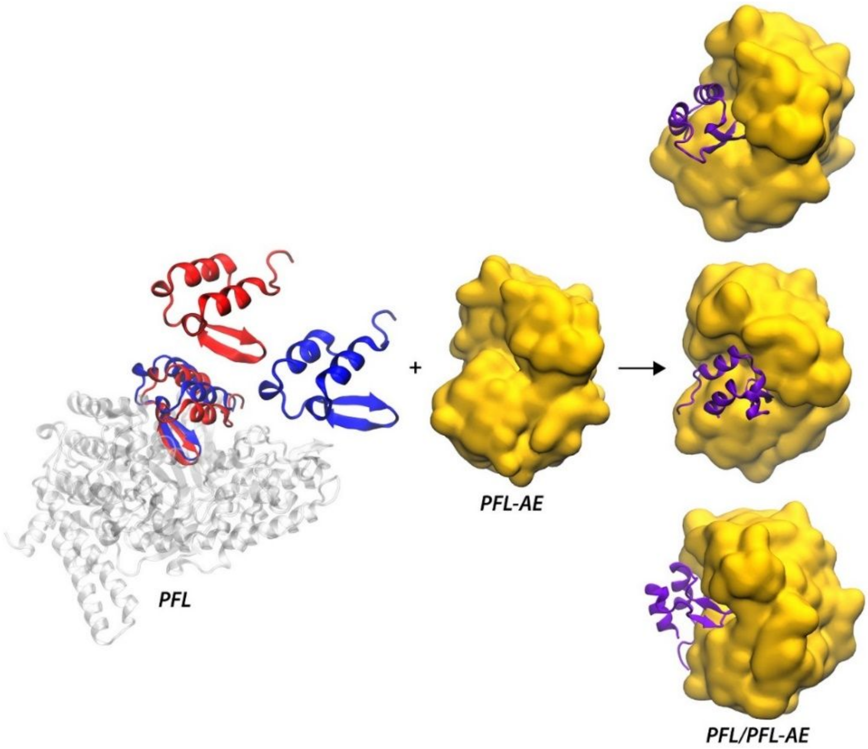

Figure S1 Depiction of general protein-protein docking protocol resulting in the three best-ranked protein-protein docking pose between C-terminus of PFL (purple ribbons) and PFL-AE (yellow isosurface). Initial docking protocols started with the preparation of input PDB files of the larger PFL-AE receptor (minimized crystal structure) and closed or partially open conformation of the entire PFL protein from long term MD simulations. No complete opening of PFL (and thus Gly exposure) could be observed in any of the performed MD simulations which is the main reason that no full PFL/PFL-AE docking results were able to resemble conformations in which Gly loop binds close to the active site of PFL-AE that is associated with the enzyme activation complexes. However, since significant structural changes in the C-terminus region could be observed, closed (red ribbons) and partially open (blue ribbons) conformation of the truncated C-terminus of PFL (residues 711-759) were extracted and treated as a ligand for protein-protein docking resulting in better structures, including the three best structures above that resemble the best candidates. To select these most suitable structures of the complex for the activation, we first selected first ten best candidates out of thirty docking structures typically obtained by ClusPro based on their binding affinity. Out of those many poses had to be rejected because the Gly loop was buried too deep in the active site (due to disregarding the presence of the FeS cluster and SAM during docking) that would have led to substantial overlap and clashes in the final structure. Then we narrowed down the selection even more by visual inspection of the complexes and the superposition with the bound peptide. This led to the best three matching/performance top scores which were selected for MD simulations. To prepare PFL-AE for docking SAM, FeS cluster, ions, and water was deleted from the PDB file. Protein-protein docking was carried out with the ClusPro 2.0 server including the definition of attractive and repulsive residues based on the previously defined and analysed interactions from MD simulations of PFL-AE with Gly peptide. The PFL-AE receptor attraction residues chosen were 8, 16, 38, 37 and 208 and the C-terminus ligand attraction residues were 733, 734 and 736. We also defined the receptor repulsion residues 166, 104, 76, 129, 131, 168, 41, 36, 42, 29, 33 and 35. Input PDB files of the PFL-AE receptor and the C-terminus ligand contained only polar hydrogens and the output complexes were classified in four categories: Balanced, Electrostatic-favoured, Hydrophobic-favoured and VdW+Electrostatic.

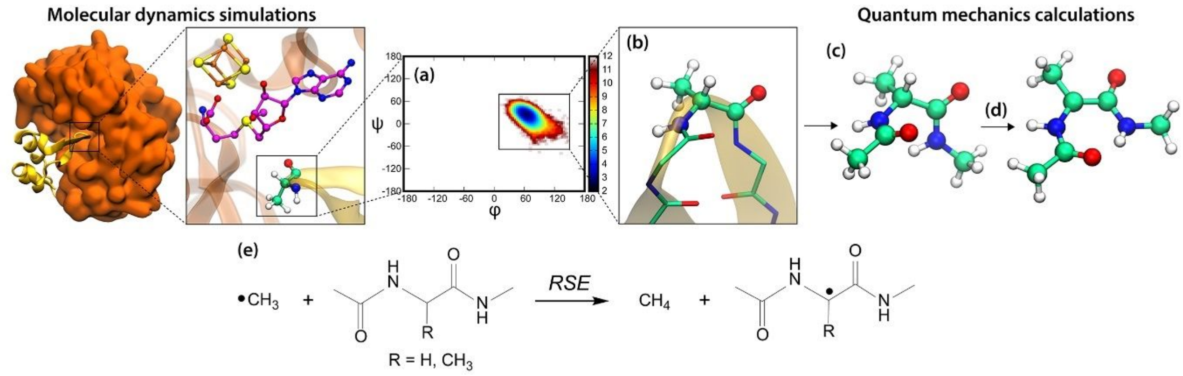

Figure S2 Protocol for radical stabilisation energy (RSE) calculations. (a) Ramachandran plots of the central Gly and (*R*)-Ala have been calculated using snapshots from molecular dynamics (MD) simulations of C-terminus in water and bound to PFL-AE and presented as free energy landscape (relative scale given in k_BT) defined by $W_i/k_BT = -\ln(N_i/N_{tot})$ where N_{tot} is the total number of configurations in each system (N_{tot} = 300 000 and T = 300 K). An equal number of bins (100) has been specified for each dimension. (b) Representative MD structures of the central Gly and (*R*)-Ala were extracted from simulations of C-terminus in water and bound to PFL-AE. (c) Model dipeptides were constructed by capping a central Gly and (*R*)-Ala with acetyl and *N*-Methyl amide groups and optimised in the gas phase using quantum mechanics (QM). (d) Pro-(*S*) hydrogen atom has been removed from the initial structure and newly constructed radical has been further optimised with QM. All RSE calculations have been performed following the isodesmic reaction shown in the panel (e) using the G3(MP2)-RAD method.

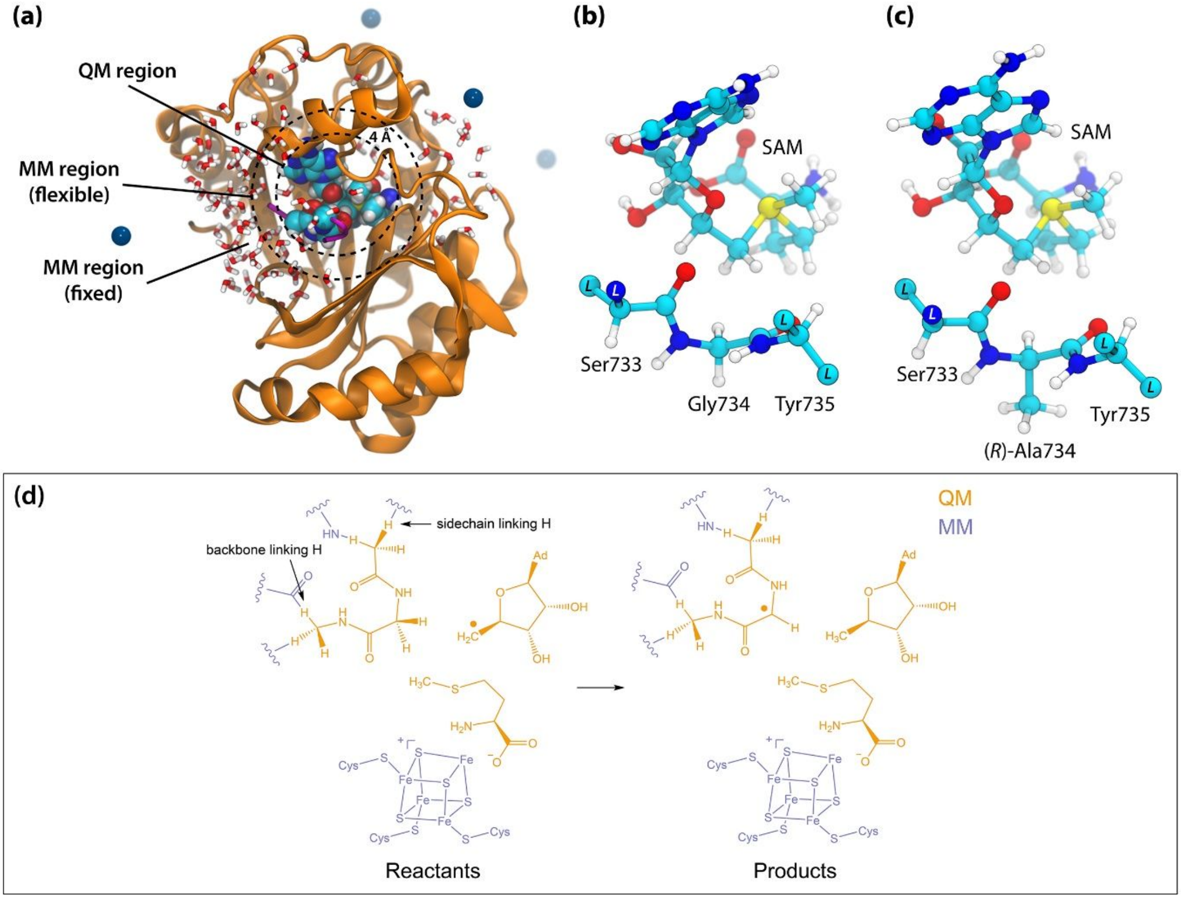

Figure S3 QM/MM calculations setup. (a) The QM/MM model system (two-layer ONIOM). The QM region as well as all MM residues that are found within 4 Å from the QM region are allowed to move during the optimisation. The rest of the protein, solvent molecules and ions are kept fixed during the optimisation and treated with MM. The QM part of the QM/MM model used in the calculations of the Gly- and (*R*)-Ala-containing systems are shown in the panels (b) and (c), respectively. The atoms treated with QM are shown in a ball-and-stick representation, while the rest of the protein is omitted for clarity. The QM region encompasses SAM, the central Gly or (*R*)-Ala residue as well as the additional part of the neighbouring Ser733 and Tyr735 (68 and 71 QM atoms in total including link H-atoms for the systems containing the central Gly and (*R*)-Ala, respectively; the charge of the QM system was -1, spin ½). The label *L* indicates the atoms replaced by hydrogen link-atoms. (d) The picture of the PFL-AE active site before and after the H-atom-transfer with QM region of the QM/MM model shown in orange and the MM region shown in grey.

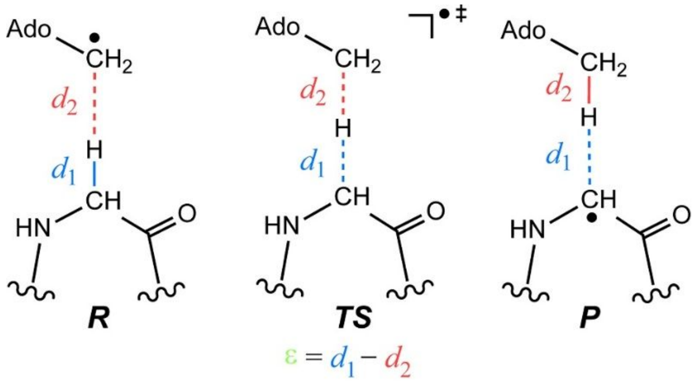

Figure S4 Definition of reaction coordinate ε for H-atom transfer between Gly or (*R*)-Ala and 5'-dAdo$^{\bullet}$ through distances d_1 and d_2.

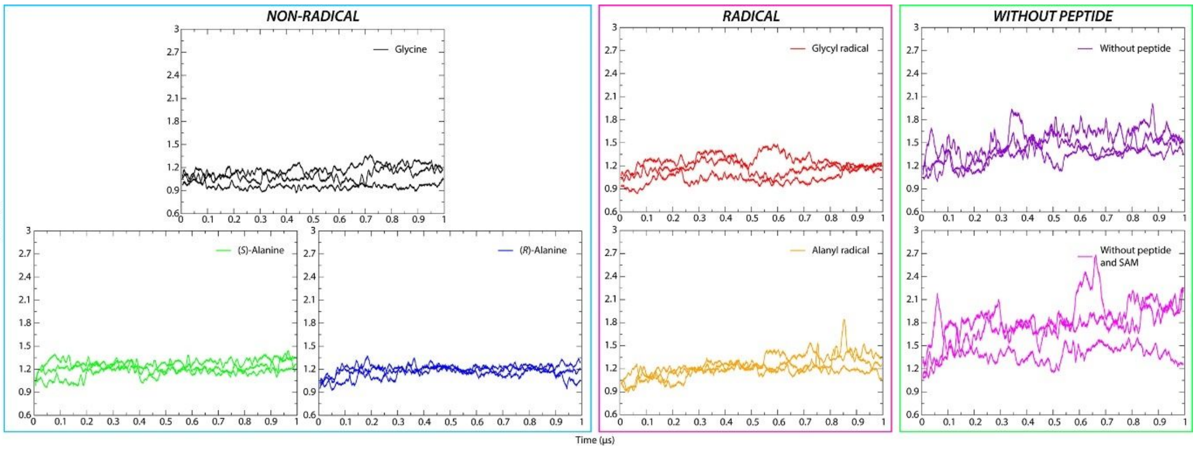

Figure S5 RMSD of protein backbone (N, C_{α}, C) calculated from the initial crystal structure of PFL-AE in the presence and the absence of peptide from 3 µs MD simulations. Note that all systems contain peptide with different central residues.

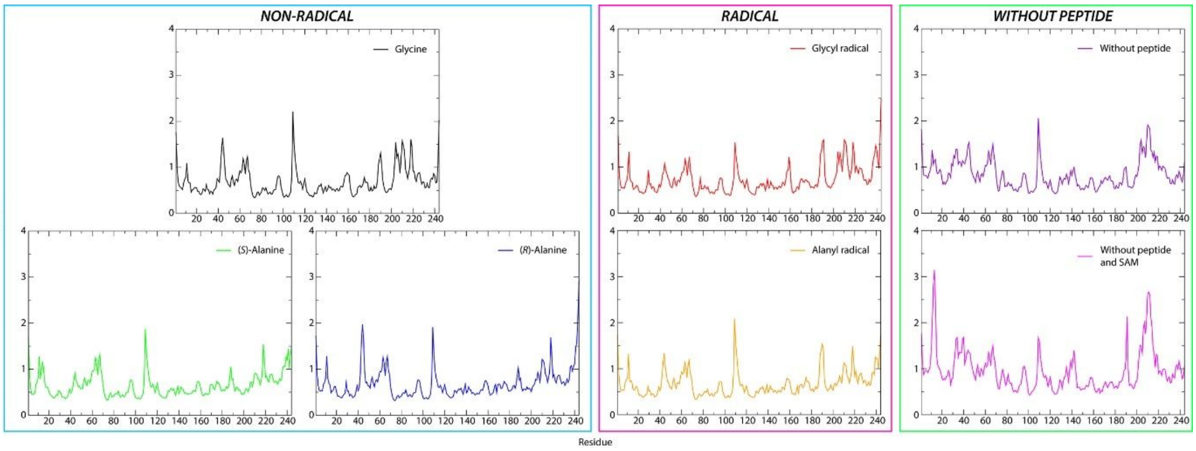

Figure S6 RMSF of protein backbone (N, C_{α}, C) calculated from the initial crystal structure of PFL-AE from 3 µs MD simulations. Note that all performed simulations are averaged to obtain the unique RMSF profile for each of the systems.

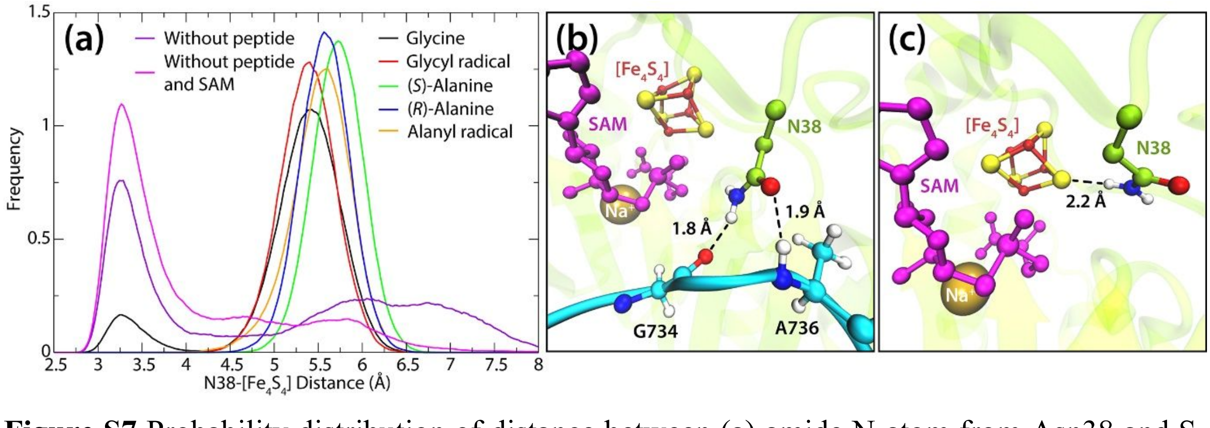

Figure S7 Probability distribution of distance between (a) amide N-atom from Asn38 and S-atom from the iron-sulfur cluster. Snapshots of the interactions between Asn38 and (b) peptide or (c) [Fe_4S_4] cluster taken from MD simulations of PFL-AE in the presence and absence of peptide, respectively. Note that each peptide contains different central residues. Data is collected from 3 µs MD simulations.

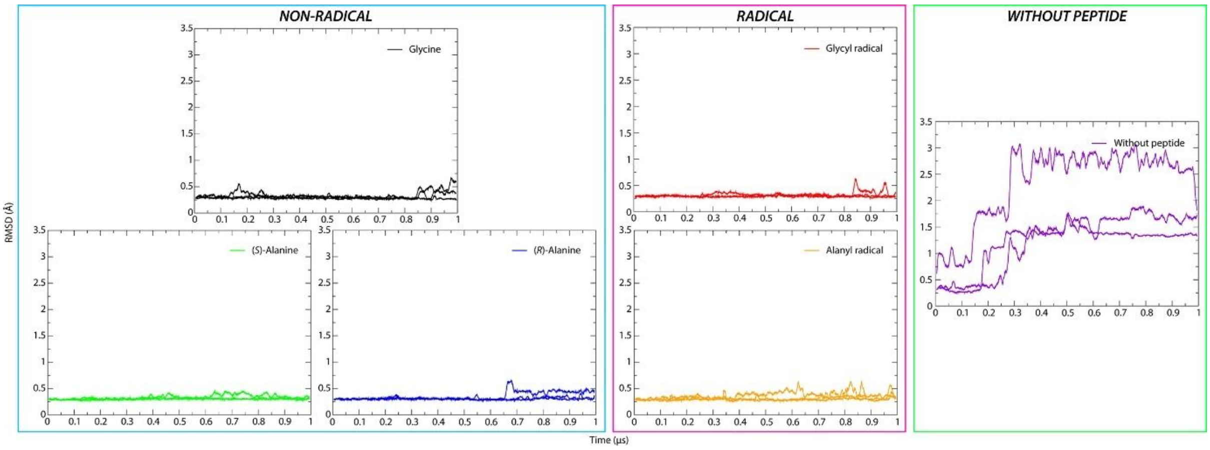

Figure S8 RMSD of SAM non-hydrogen atoms. All deviations are calculated from the initial crystal structure. Data is collected for all model systems containing PFL-AE from 3 µs MD simulations.

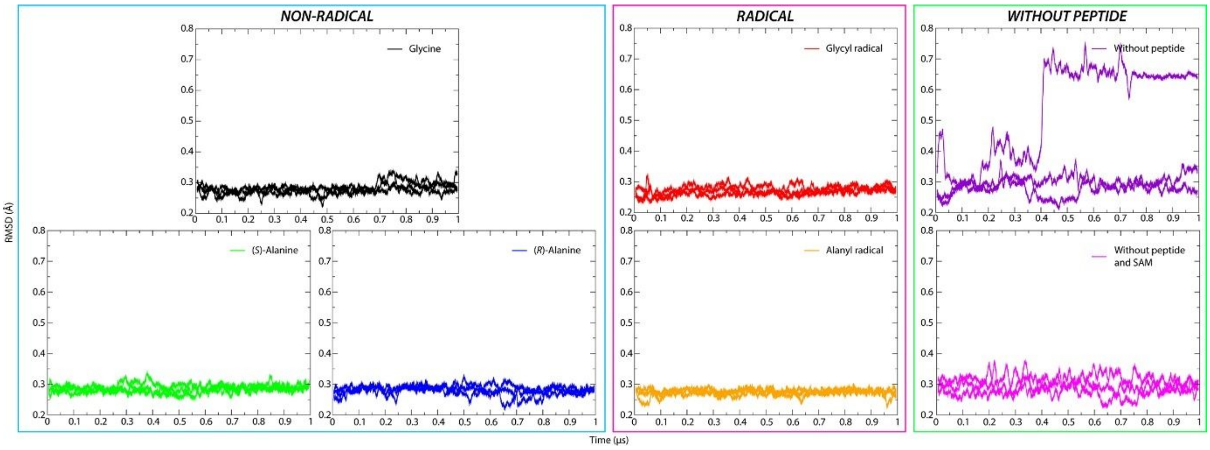

Figure S9 RMSD of [Fe_4S_4] cluster. All deviations were calculated from the initial crystal structure. Data is collected for all model systems containing PFL-AE from 3 µs MD simulations.

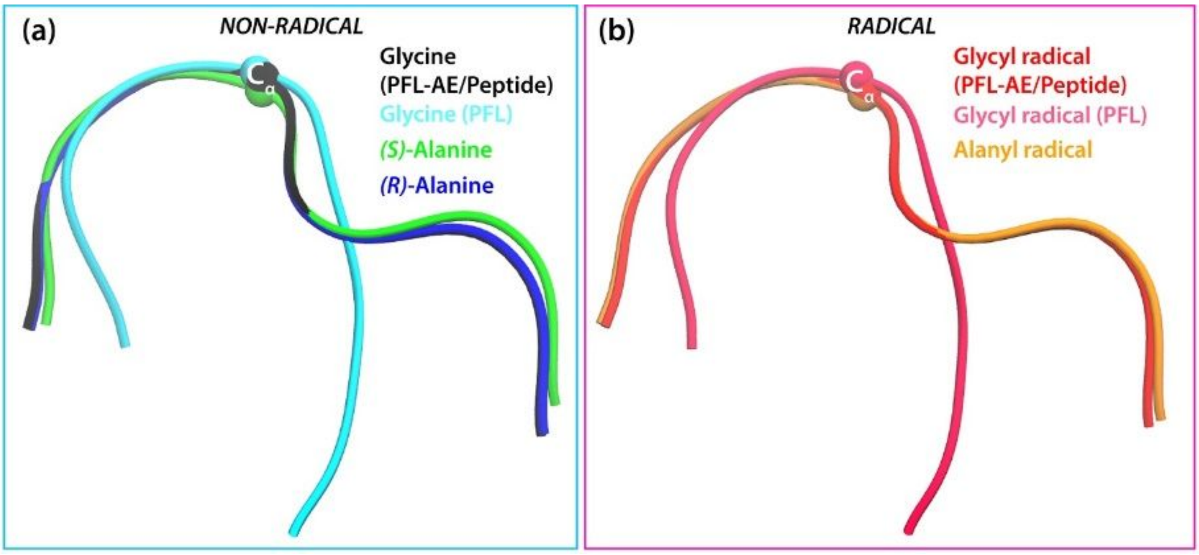

Figure S10 Average structures of (a) non-radical- and (b) radical-containing peptide bound to PFL-AE and Gly radical domain in PFL. The average conformation is obtained by collecting the snapshots from 3 μs MD simulations. All snapshots are superimposed to the backbone of central residue 734.

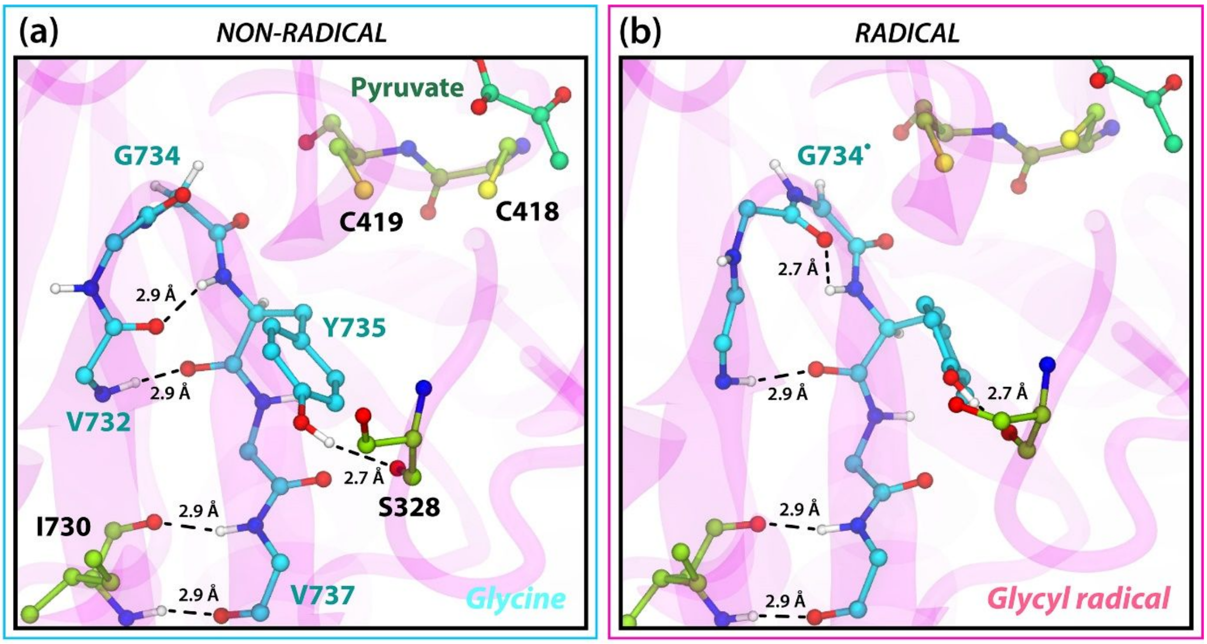

Figure S11 Average structures of (a) Gly- and (b) Gly radical-containing Gly radical domain in PFL showing important interactions. The average conformation is obtained by collecting snapshots from 3 μs MD simulations.

Table S1 Hydrogen bonds between Gly radical domain and the rest of PFL from 3 μs MD simulations. Only intermolecular interactions with frequency larger than 10% are listed.

		Glycine			
Acceptor	Hydrogen atom donor	Heteroatom donor	Fraction (%)	Average distance (Å)	Average angle (°)
		**V732**			
Y735 O	**V732 H**	**V732 N**	**79**	2.85	185.20
**V732 O**	Y735 H	Y735 N	**76**	2.85	158.82
		**S733**			
**S733 OG**	R753 HH11	R753 NH1	**33**	2.85	161.12
		**G734**			
T666 OG1	**G734 H**	**G734 N**	**46**	2.88	148.61
		**Y735**			
S328 O	**Y735 HH**	**Y735 OH**	**98**	2.70	164.51
**Y735 O**	V732 H	V732 N	**79**	2.85	158.20
V732 O	**Y735 H**	**Y735 N**	**76**	2.85	158.82
**Y735 O**	N706 HD22	N706 ND2	**39**	2.89	162.48
		**A736**			
		-			
		**V737**			
**V737 O**	I730 H	I730 N	**55**	2.89	163.18
I730 O	**V737 H**	**V737 N**	**27**	2.92	161.99
		Glycyl radical			
		**V732**			
Y735 O	**V732 H**	**V732 N**	**63**	2.86	160.23
**V732 O**	Y735 H	Y735 N	**28**	2.84	157.68

**V732 O**	S733 HG	S733 OG	**19**	2.69	155.54
			**S733**		
**S733 O**	Y735 H	Y735 N	**19**	2.73	143.14
V732 O	**S733 HG**	**S733 OG**	**19**	2.69	155.54
I606 O	**S733 HG**	**S733 OG**	**18**	2.74	156.70
**S733 OG**	S664 HG	S664 OG	**11**	2.78	160.73
			**G734•**		
T666 OG1	**G734 H**	**G734 N**	**12**	2.89	152.31
**G734 O**	W333 HE1	W333 NE1	**11**	2.88	162.89
			**Y735**		
S328 O	**Y735 HH**	**Y735 OH**	**92**	2.72	163.52
**Y735 O**	V732 H	V732 N	**63**	2.86	160.23
V732 O	**Y735 H**	**Y735 N**	**28**	2.84	157.68
S733 O	**Y735 H**	**Y735 N**	**19**	2.73	143.14
**Y735 O**	N706 HD22	N706 ND2	**11**	2.88	163.89
			**A736**		
			-		
			**V737**		
**V737 O**	I730 H	I730 N	**51**	2.90	161.92
I730 O	**V737 H**	**V737 H**	**48**	2.90	160.60

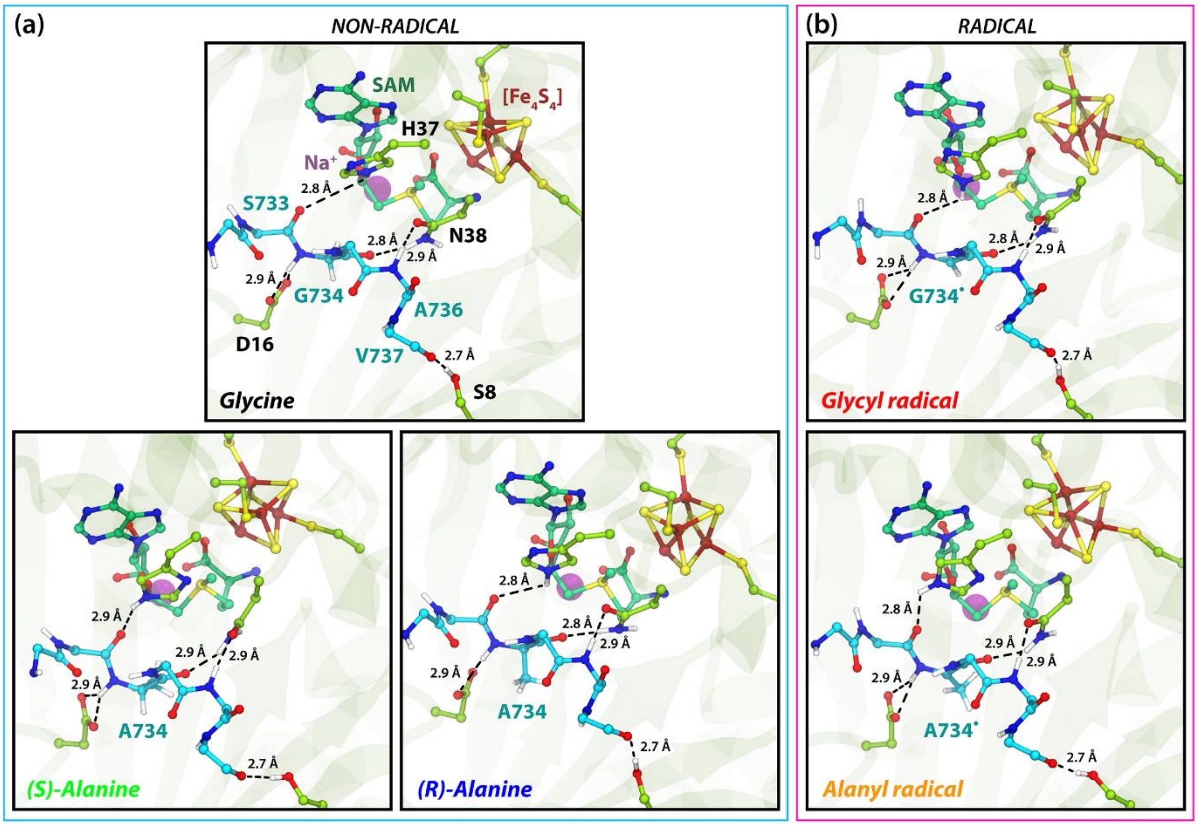

Figure S12 Average structures of (a) non-radical- and (b) radical-containing peptide bound to PFL-AE showing important interactions. The average conformation is obtained by collecting snapshots from 3 μs MD simulations.

Table S2 Hydrogen bonds between model peptide and PFL-AE from 3 μs MD simulations. Only intermolecular interactions with frequency larger than 10% are listed.

Glycine					
Acceptor	Hydrogen atom donor	Heteroatom donor	Fraction (%)	Average distance (Å)	Average angle (°)
		**V732**			
		-			
		**S733**			
**S733 O**	H37 HE2	H37 NE2	**25**	2.84	148.65
V15 O	**S733 HG**	**S733 OG**	**14**	2.77	162.26
		**G734**			
**G734 O**	N38 HD21	N38 ND2	**71**	2.84	155.63
D16 OD	**G734 H**	**G734 N**	**36**	2.87	155.39
		**Y735**			
		-			
		**A736**			
N38 OD1	**A736 H**	**A736 N**	**62**	2.86	164.05
**A736 O**	K207 HZ	K207 NZ	**12**	2.83	157.70
		**V737**			
**V737 O**	S8 HG	S8 OG	**78**	2.72	162.71
		Glycyl radical			

		**V732**			
		-			
		**S733**			
D16 OD	**S733 HG**	**S733 OG**	**22**	2.66	161.87
**S733 O**	H37 HE2	H37 NE2	**12**	2.84	147.73
		**G734˙**			
**G734 O**	N38 HD21	N38 ND2	**80**	2.84	155.08
D16 OD	**G734 H**	**G734 N**	**41**	2.86	159.98
		**Y735**			
		-			
		**A736**			
N38 OD1	**A736 H**	**A736 N**	**61**	2.88	164.38
		**V737**			
**V737 O**	S8 HG	S8 OG	**86**	2.71	161.96
		(S)-Alanine			
		**V732**			
		-			
		**S733**			
**S733 O**	H37 HE2	H37 NE2	**23**	2.85	150.40
D16 OD	**S733 HG**	**S733 OG**	**13**	2.65	163.76
		**A734**			
**A734 O**	N38 HD21	N38 ND2	**61**	2.88	158.69
D16 OD	**A734 H**	**A734 N**	**23**	2.86	155.08
		**Y735**			
		-			
		**A736**			
N38 OD1	**A736 H**	**A736 N**	**60**	2.87	162.34
		**V737**			
**V737 O**	S8 HG	S8 OG	**74**	2.71	162.29
		(R)-Alanine			
		**V732**			
		-			
		**S733**			
D16 OD	**S733 HG**	**S733 OG**	**32**	2.64	163.33
		**A734**			
**A734 O**	N38 HD21	N38 ND2	**81**	2.82	149.40
D16 OD	**A734 H**	**A734 N**	**31**	2.87	155.94
		**Y735**			
		-			
		**A736**			
N38 OD1	**A736 H**	**A736 N**	**61**	2.87	163.84
		**V737**			
**V737 O**	S8 HG	S8 OG	**76**	2.71	162.66
		Alanyl radical			
		**V732**			

		-			
		**S733**			
**S733 O**	H37 HE2	H37 NE2	**33**	2.82	149.40
D16 OD	**S733 HG**	**S733 OG**	**18**	2.66	162.84
		**A734•**			
**A734 O**	N38 HD21	N38 ND2	**68**	2.86	156.29
D16 OD	**A734 H**	**A734 N**	**27**	2.89	158.12
		**Y735**			
		-			
		**A736**			
N38 OD1	**A736 H**	**A736 N**	**67**	2.87	163.63
		**V737**			
**V737 O**	S8 HG	S8 OG	**73**	2.71	162.05

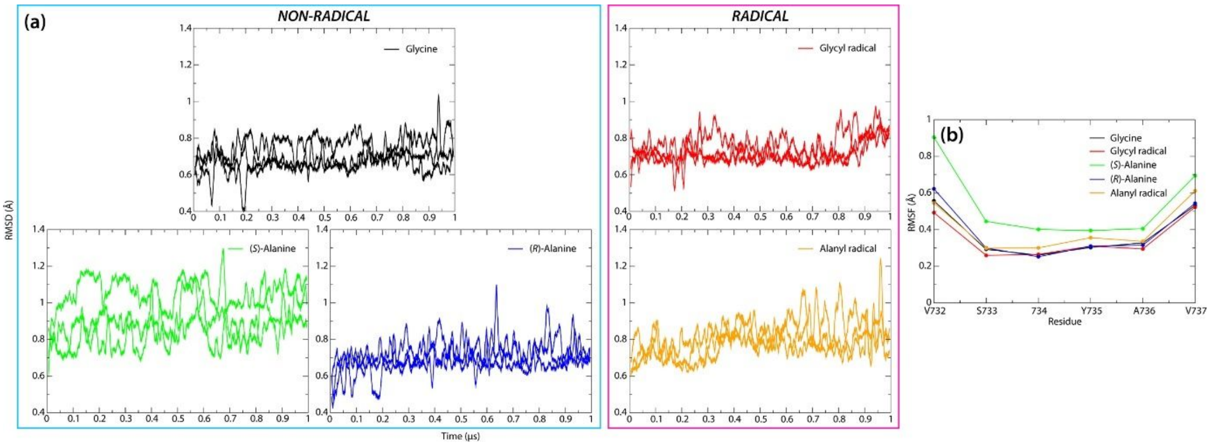

Figure S13 (a) RMSD and (b) RMSF of peptide backbone (N, C_α, C) calculated from the initial crystal structure of the peptide in complex with PFL-AE from 3 μs MD simulations. Note that each peptide contains different central residues.

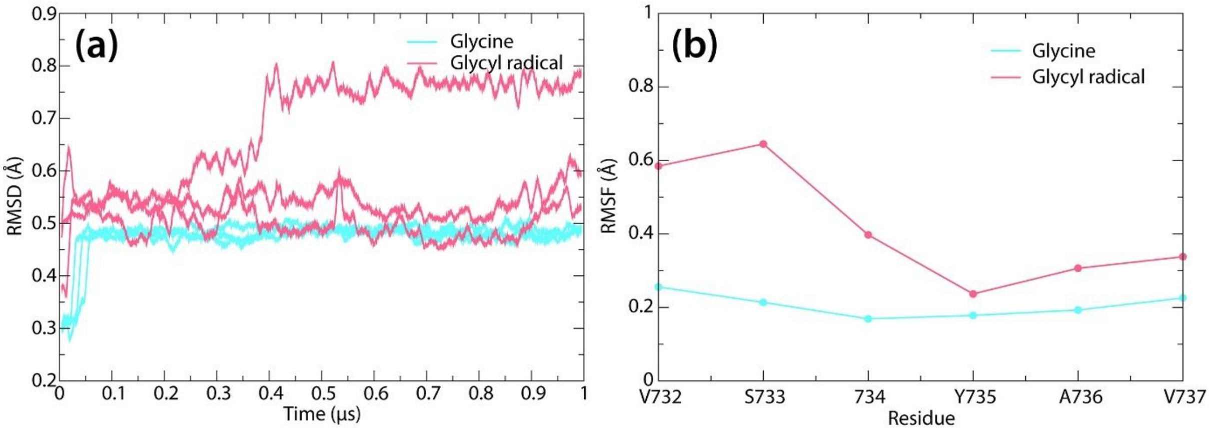

Figure S14 (a) RMSD and (b) RMSF of Gly loop backbone (N, C_α, C) calculated from the initial crystal structure of PFL with non-radical- or radical-containing Gly radical domain from 3 μs MD simulations.

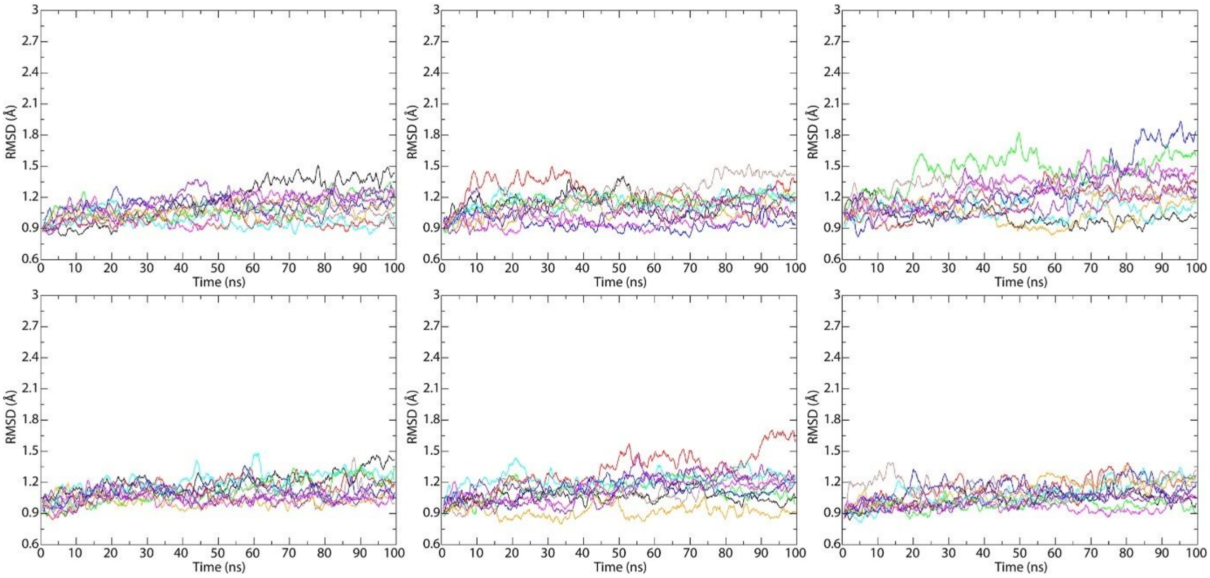

Figure S15 RMSD of PFL-AE backbone (N, C_α, C) calculated from the initial crystal structure of PFL-AE from 10 independent MD simulations (100 ns each) of three docking complex between PFL-AE and C-terminus. Note that each system contains C-terminus with Gly (upper) or (*R*)-Ala (lower) central residue.

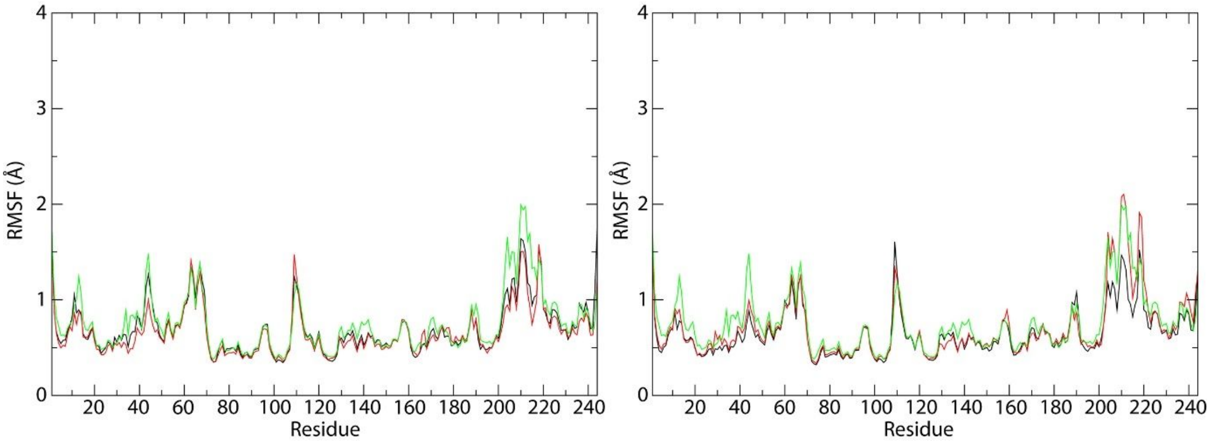

Figure S16 RMSF of PFL-AE backbone (N, C_α, C) calculated from the initial crystal structure of PFL-AE from 10 independent 100 ns MD simulations of three docking complex between PFL-AE and C-terminus. Note that all performed simulations are averaged to obtain the unique RMSF profile for each of the systems. Systems contain C-terminus with Gly (left) or (*R*)-Ala (right).

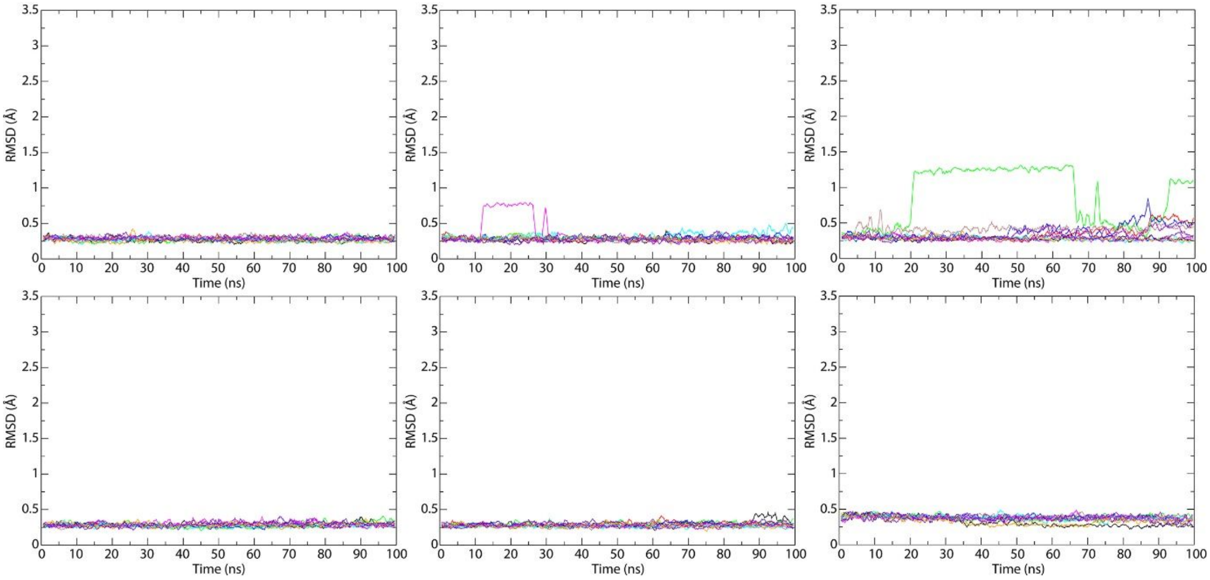

Figure S17 RMSD of SAM non-hydrogen atoms. All deviations are calculated from the initial crystal structure. Data is collected for both model systems (Gly (upper) and (*R*)-Ala (lower)) containing PFL-AE in complex with C-terminus (three different docking poses) from 10 independent 100 ns MD simulations.

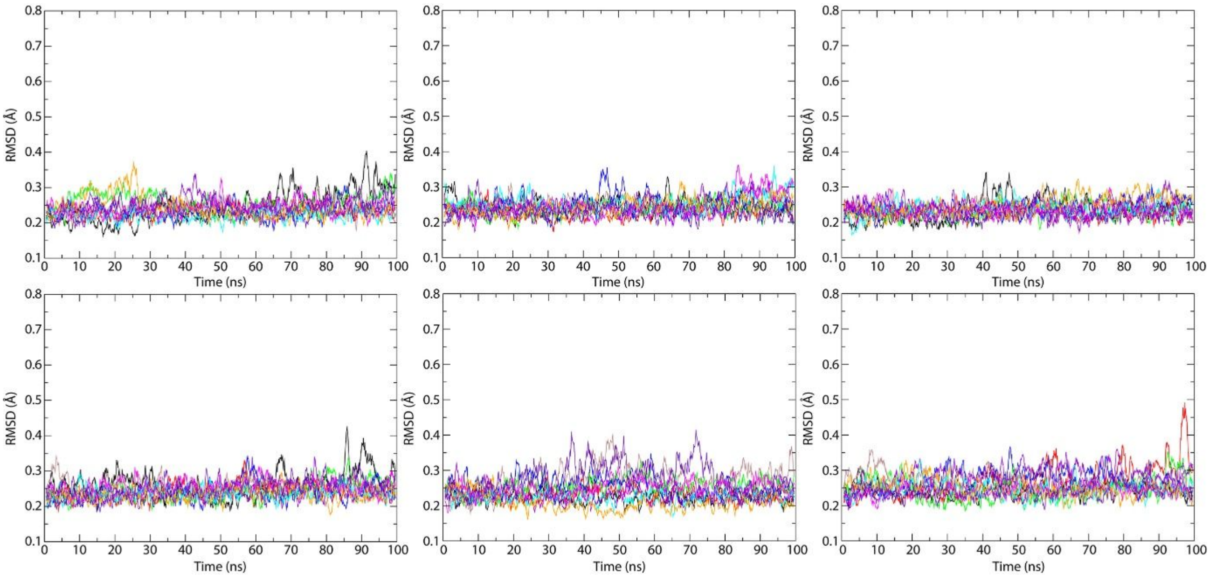

Figure S18 RMSD of [Fe_4S_4] cluster. All deviations were calculated from the initial crystal structure. Data is collected for both model systems (Gly (upper) and (*R*)-Ala (lower)) containing PFL-AE in complex with C-terminus (three different docking poses) from 10 independent 100 ns MD simulations.

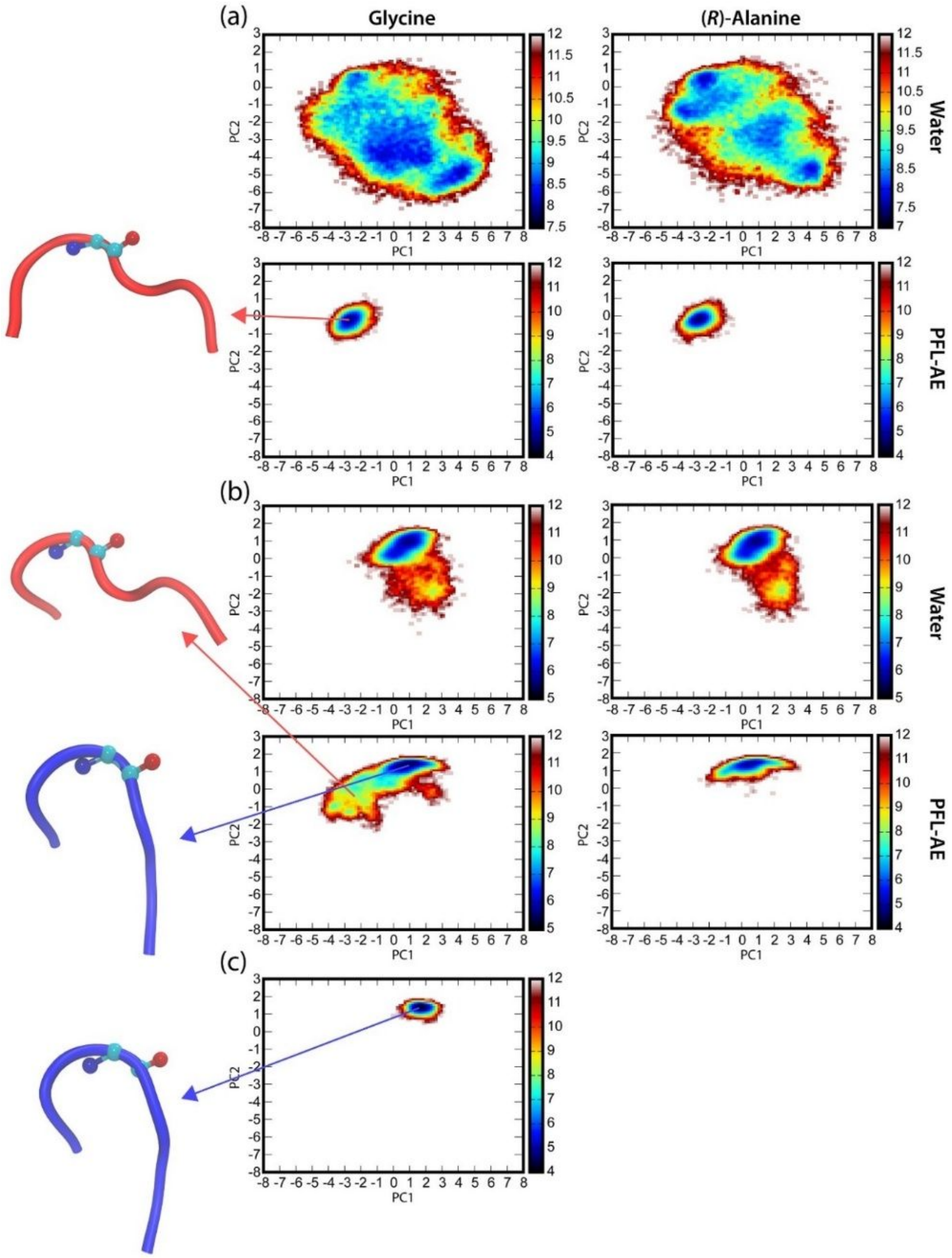

Figure S19 Free energy landscape along PC1 and PC2 constructed using 30 000 snapshots from 3 μs MD simulations of (a) peptides (Gly (left) and (*R*)-Ala (right)) in water (upper) and bound to PFL-AE (lower), (b) C-terminus loop residues (Gly (left) and (*R*)-Ala (right)) in water (upper) and bound to PFL-AE (lower) and (c) PFL loop residues. Principal components were created considering backbone atoms (N, C_α, C and O) of six loop residues. The free energy (k_BT) heatmap is defined by $W_i/k_BT = -\ln(N_i/N_{tot})$ where N_{tot} is the total number of configurations in each system (N_{tot} = 30 000 and T = 300 K). An equal number of bins (100) has been specified for each dimension. Representative structure of the loop (with central Gly and (*R*)-Ala backbone atoms) is shown.

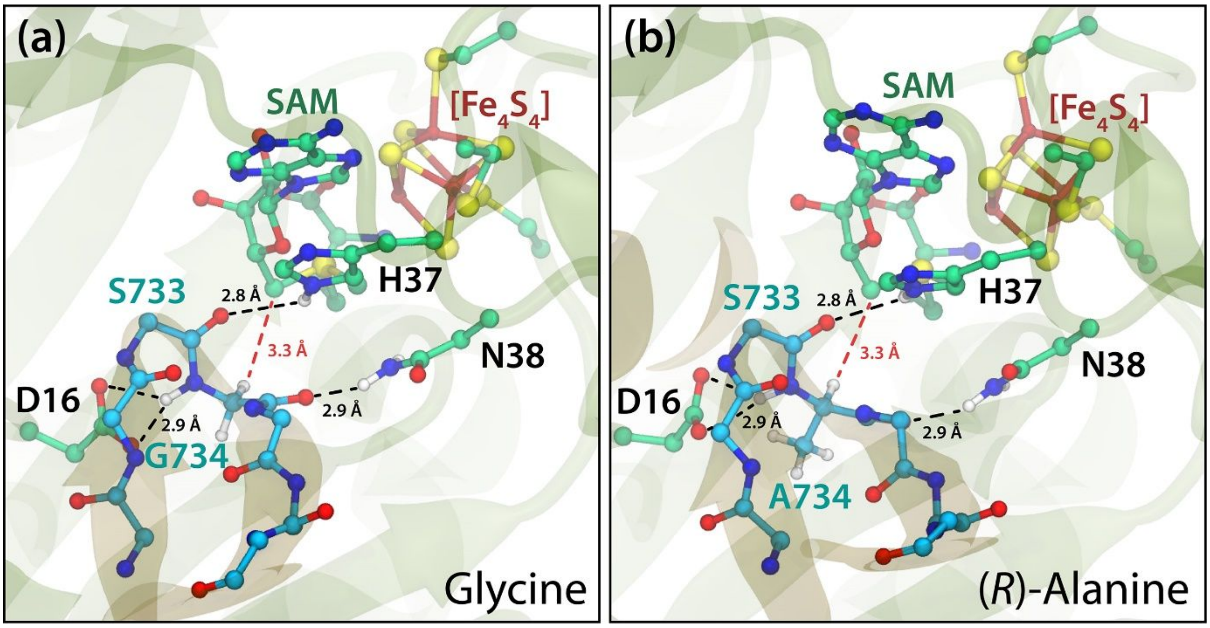

Figure S20 Representative structure of C-terminus with central (a) Gly and (b) (*R*)-Ala bound to PFL-AE extracted from 3 µs MD simulations. Crucial interactions with Asp16, His37, Asn38 are highlighted as black and with SAM as red dashed lines.

Table S3 Hydrogen bonds between C-terminus of PFL and PFL-AE from 3 µs MD simulations. Only intermolecular interactions with frequency larger than 10% are listed.

			Glycine		
Acceptor	Hydrogen atom donor	Heteroatom donor	Fraction (%)	Average distance (Å)	Average angle (°)
			**V732**		
			-		
			**S733**		
**S733 O**	H37 HE2	H37 NE2	**32**	2.84	150.24
D16 OD	**S733 HG**	**S733 OG**	**13**	2.67	163.85
			**G734**		
**G734 O**	N38 HD21	N38 ND2	**42**	2.85	159.29
D16 OD	**G734 H**	**G734 N**	**18**	2.86	157.55
			**Y735**		
			-		
			**A736**		
			-		
			**V737**		
			-		
			(R)-Alanine		
			**V732**		
			-		
			**S733**		
**S733 O**	H37 HE2	H37 NE2	**20**	2.86	146.79
			**A734**		
**A734 O**	N38 HD21	N38 ND2	**58**	2.85	158.34

D16 OD	**A734 H**	**A734 N**	**20**	2.88	157.31
			**Y735**		
			-		
			**A736**		
			-		
			**V737**		
			-		

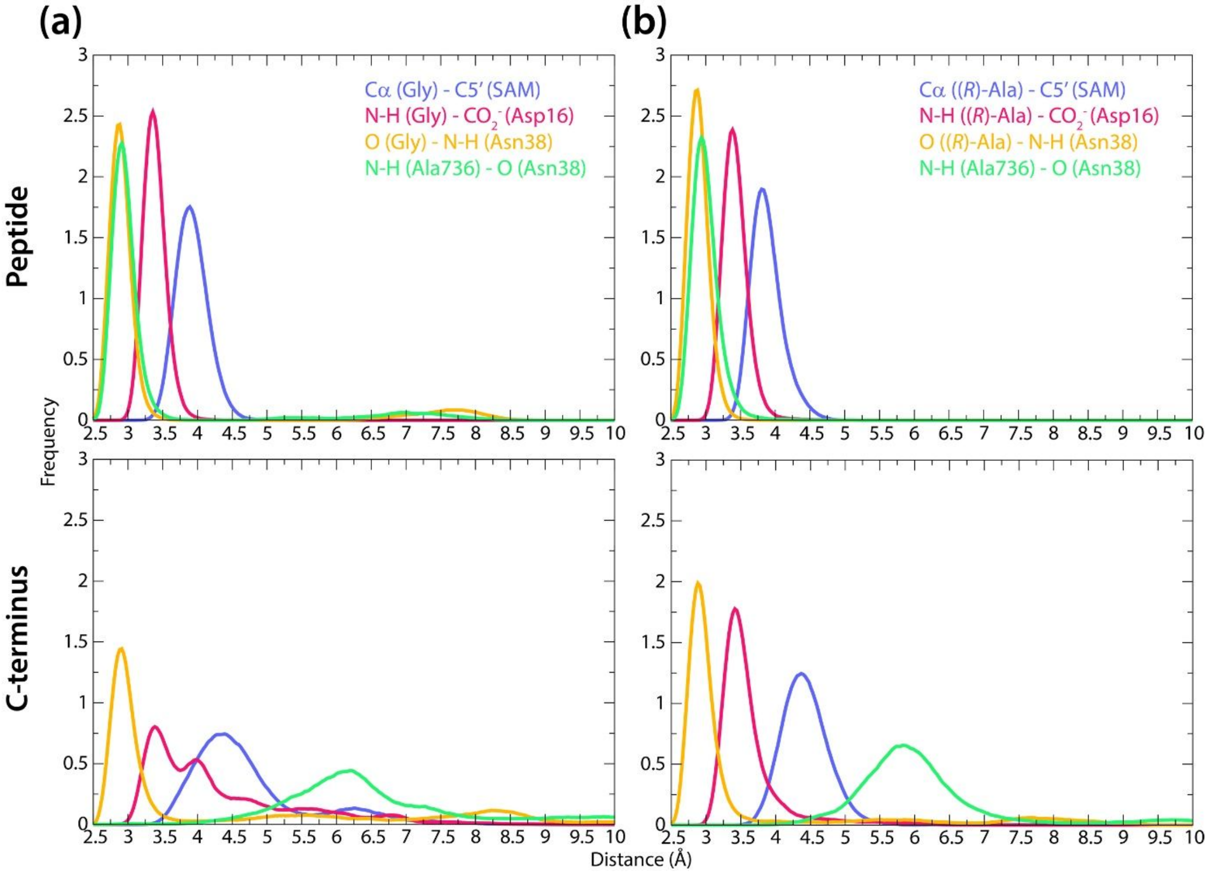

Figure S21 Probability distribution of key interactions between peptide (upper) or C-terminus (lower) and PFL-AE during MD simulations of PFL-AE in the presence of (a) Gly- and (b) (*R*)-Ala-containing peptide or C-terminus. The data was collected from 3 μs MD simulations of peptide or C-terminus bound to PFL-AE.

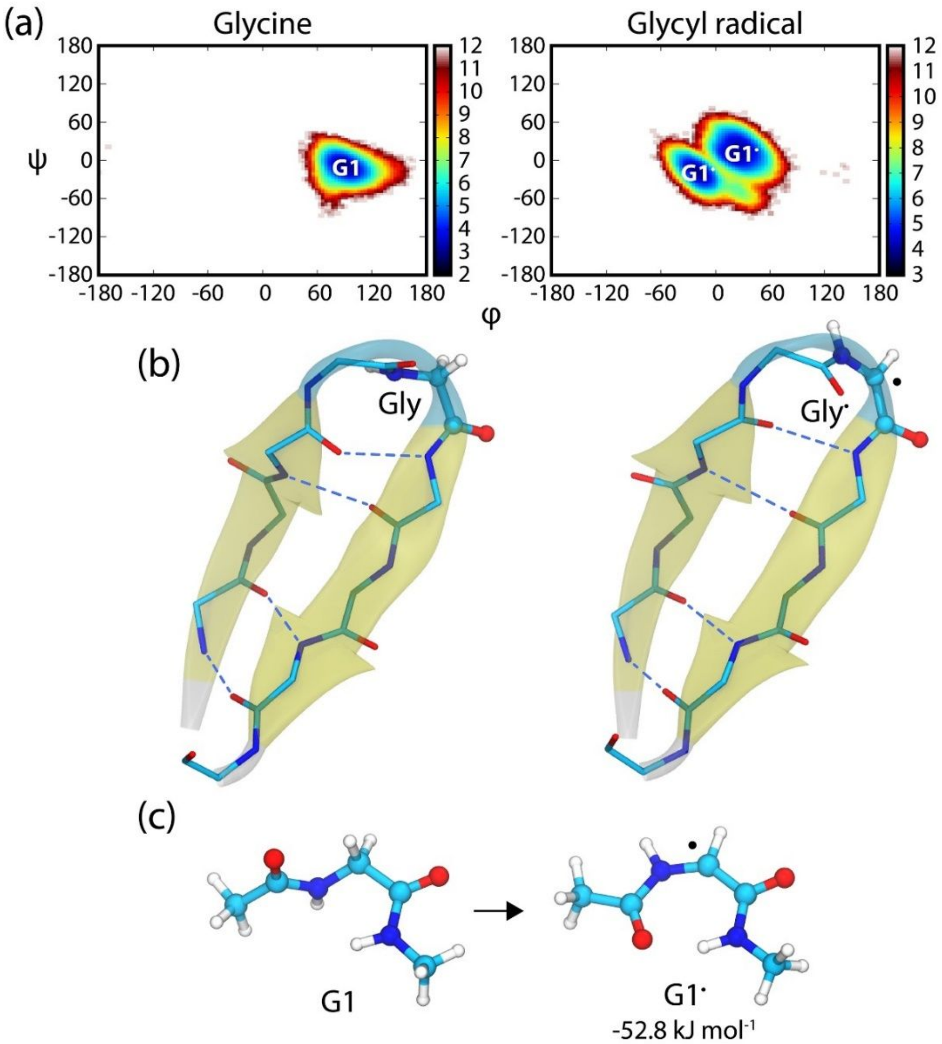

Figure S22 (a) Ramachandran plots of the non-radical and radical Gly734 residue from MD simulations of PFL shown as free energy landscape. The free energy (relative values given in k_BT) heatmap is defined by $W_i/k_BT = -\ln(N_i/N_{tot})$ where N_{tot} is the total number of configurations in each system (N_{tot} = 300 000 and T = 300 K). An equal number of bins (100) has been specified for each dimension. (b) Cartoon representation of a β-hairpin motif with glycine and glycyl radical located in the turn. The stabilising hydrogen bonds between the two antiparallel beta sheets are shown as blue dashed lines. (c) Optimised dipeptide models of the dominant conformer G1 and G1$^{\bullet}$ used in the radical stabilisation energy calculations. The RSE shown in panel (c) was calculated with the G3(MP2)-RAD method.

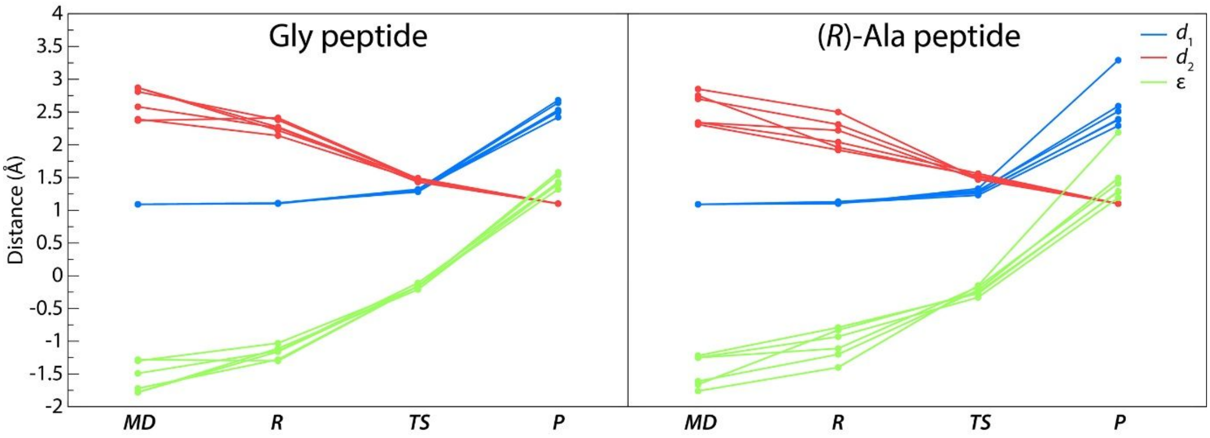

Figure S23 Values of the reaction coordinate ε and distances d_1 and d_2 in MD snapshots and QM/MM optimised structures during the H-atom transfer between 5'-dAdo• and peptide containing central Gly or (*R*)-Ala.

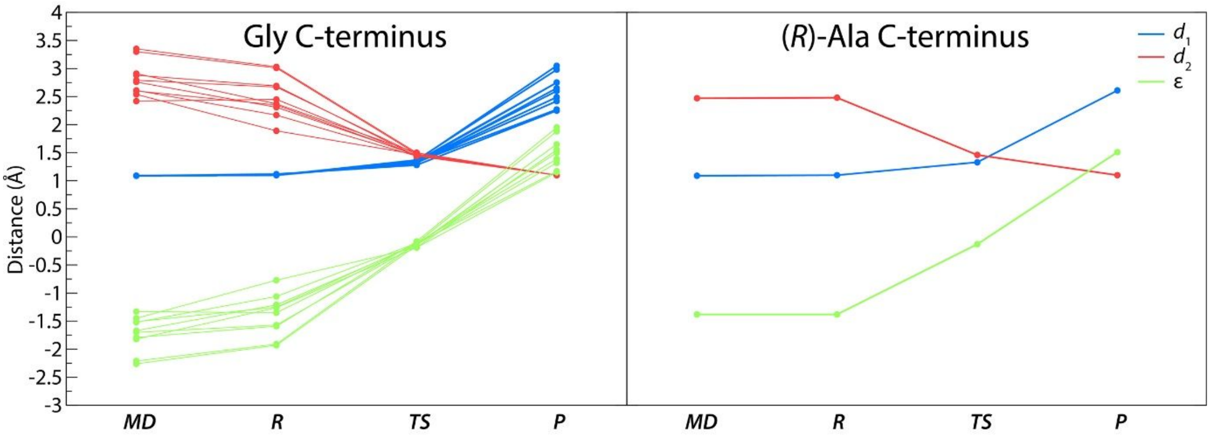

Figure S24 Values of the reaction ε and distances d_1 and d_2 in MD snapshots and QM/MM optimised structures during the H-atom transfer between 5'-dAdo• and C-terminus containing central Gly or (*R*)-Ala.

Table S4 The sum of G3(MP2)-RAD and scaled zero-point energies of molecular species used in RSE calculations.

Structure	E (hartree)
**CH_4**	-40.4302475
**$CH_3^•$**	-39.7653710
**G1**	-455.9420039
**G2**	-455.9460821
**G3**	-455.9420073
**G4**	-455.9449192
**G1$^•$**	-455.2972388
**G2$^•$**	-455.2988745
**G3$^•$**	-455.3093384
**A1**	-495.1900049
**A2**	-495.1952513
**A3**	-495.1931216
**A1$^•$**	-494.5482005
**A2$^•$**	-494.5551381

Table S5 The sum of ONIOM and zero-point energies for reactants, transition states and products calculated at the ONIOM[TPSS+D3/def2-TZVP:Amber] level of theory. The lowest harmonic frequencies ω_i obtained from vibrational analysis of transition states with the ONIOM[TPSS+D3/def2-SVP:Amber] method.

	E (hartree)					
	**Gly Peptide**					
**Structure**	**1**	**2**	**3**	**4**	**5**	**6**
**R**	-2152.9572787	-2153.1536223	-2153.4567982	-2153.4019025	-2153.2197118	-2153.3274404
**TS**	-2152.9540985	-2153.1443892	-2153.4484854	-2153.3992111	-2153.2130596	-2153.3200411
**P**	-2152.9703972	-2153.1712807	-2153.4699269	-2153.4166319	-2153.2341051	-2153.3419032
**ω_i (cm^{-1})**	1209.0685i	1478.7933i	1384.0082i	1154.0960i	1334.0542i	1366.2637i

	E (hartree)					
	**(*R*)-Ala Peptide**					
**Structure**	**1**	**2**	**3**	**4**	**5**	**6**
**R**	-2192.6705782	-2192.4161758	-2192.4557026	-2192.5904587	-2192.8240066	-2192.5462973
**TS**	-2192.6675293	-2192.4098245	-2192.4526447	-2192.5727400	-2192.8198802	-2192.5404544
**P**	-2192.6871996	-2192.4283817	-2192.4787835	-2192.6016875	-2192.8464721	-2192.5629383
**ω_i (cm^{-1})**	595.6005i	1248.0056i	944.2597i	1500.0722i	1055.4103i	1143.6820i

	E (hartree)					
	**Gly C-terminus**					**(*R*)-Ala C-terminus**
**Structure**	**1**	**2**	**3**	**4**	**5**	**1**
**R**	-2155.6295255	-2156.0157200	-2155.6698587	-2155.9592335	-2156.2038615	-2195.2628607
**TS**	-2155.6193597	-2155.9813139	-2155.6481641	-2155.9461953	-2156.2008297	-2195.2473065
**P**	-2155.6375010	-2156.0265499	-2155.6787054	-2155.9791829	-2156.2187126	-2195.2703867
**ω_i (cm^{-1})**	1503.6241i	1688.8776i	1591.7744i	1367.7224i	1177.8735i	1476.6053i
**Structure**	**6**	**7**	**8**	**9**	**10**	
**R**	-2155.8619820	-2155.7494994	-2156.1396994	-2156.1615065	-2155.9957198	
**TS**	-2155.8545975	-2155.7356001	-2156.1256536	-2156.1504399	-2155.9755863	
**P**	-2155.8682517	-2155.7615319	-2156.1422634	-2156.1695612	-2155.9966659	
**ω_i (cm^{-1})**	1445.5753i	1472.1420i	1480.5918i	1424.1475i	1635.6631i	
